# Persistence and selection of an expanded B-cell clone in the setting of rituximab therapy for Sjögren’s syndrome

**DOI:** 10.1186/ar4481

**Published:** 2014-02-11

**Authors:** Uri Hershberg, Wenzhao Meng, Bochao Zhang, Nancy Haff, E William St Clair, Philip L Cohen, Patrice D McNair, Ling Li, Marc C Levesque, Eline T Luning Prak

**Affiliations:** 1 School of Biomedical Engineering, Science and Health Systems, 711 Bossone Building, Drexel University, 3141 Chestnut Street, Philadelphia, PA 19104, USA; 2 Department of Microbiology and Immunology, College of Medicine, 2900 Queen Lane, Philadelphia, PA 19129, USA; 3 Department of Pathology and Laboratory Medicine, Perelman School of Medicine, University of Pennsylvania, 405B Stellar Chance Labs, 422 Curie Boulevard, Philadelphia, PA 19104, USA; 4 Duke University Medical Center, 3874 200 Trent Drive, Durham, NC 27710, USA; 5 Section of Rheumatology and Temple Autoimmunity Center, Temple University School of Medicine, 3322 North Broad Street, Philadelphia, PA 19140, USA; 6 Division of Rheumatology and Clinical Immunology, University of Pittsburgh School of Medicine, 3500 Terrace Street, BST S709, Pittsburgh, PA 15261, USA

## Abstract

**Introduction:**

Subjects with primary Sjögren’s syndrome (SjS) have an increased risk of developing B-cell lymphoma and may harbor monoclonal B-cell expansions in the peripheral blood. Expanded B-cell clones could be pathogenic, and their persistence could exacerbate disease or predispose toward the development of lymphoma. Therapy with anti-CD20 (rituximab) has the potential to eliminate expanded B-cell clones and thereby potentially ameliorate disease. This study was undertaken to identify and track expanded B-cell clones in the blood of subjects with primary SjS who were treated with rituximab.

**Methods:**

To determine whether circulating B-cell clones in subjects with primary SjS emerge or remain after B cell-depleting therapy with rituximab, we studied the antibody heavy-chain repertoire. We performed single-memory B-cell and plasmablast sorting and antibody heavy-chain sequencing in six rituximab-treated SjS subjects over the course of a 1-year follow-up period.

**Results:**

Expanded B-cell clones were identified in four out of the six rituximab-treated SjS subjects, based upon the independent amplification of sequences with identical or highly similar VH, DH, and JH gene segments. We identified one SjS subject with a large expanded B-cell clone that was present prior to therapy and persisted after therapy. Somatic mutations in the clone were numerous but did not increase in frequency over the course of the 1-year follow-up, suggesting that the clone had been present for a long period of time. Intriguingly, a majority of the somatic mutations in the clone were silent, suggesting that the clone was under chronic negative selection.

**Conclusions:**

For some subjects with primary SjS, these data show that (a) expanded B-cell clones are readily identified in the peripheral blood, (b) some clones are not eliminated by rituximab, and (c) persistent clones may be under chronic negative selection or may not be antigen-driven. The analysis of sequence variation among members of an expanded clone may provide a novel means of measuring the chronicity and selection of expanded B-cell populations in humans.

## Introduction

It has been estimated that up to 3 million adults in the US suffer from primary Sjögren’s syndrome (SjS) [1]. Primary SjS is an autoimmune disorder characterized by chronic inflammation of the salivary and lacrimal glands and the presence of antinuclear antibodies, most often of the anti-SSA(Ro) and anti-SSB (La) specificities. Patients are often middle-aged females who present with sicca symptoms, such as dry eyes and dry mouth, fatigue, and joint pain, as well as other extraglandular manifestations, including lung disease and neuropathy. In primary SjS, it is believed that both T cells and B cells contribute to disease pathogenesis. Both cell types infiltrate the salivary and other exocrine glands and show evidence of clonal expansion in the affected tissues as well as the circulation [2]. Notably, there is an increased risk of lymphoma in patients with primary SjS [3].

Why patients with primary SjS are at increased risk for lymphoma is unclear and has been the subject of several studies (reviewed in [4]). One theory is that B-cell hyperactivity in primary SjS results in the abnormal activation of autoreactive B-cells and contributes to their clonal expansion [5]. Autoreactive B-cell clones, such as the recently described CD21^dim^ population in SjS, may remain chronically activated instead of anergic in the presence of self-antigens [6]. Autoreactive B-cell clones may also have increased resistance to apoptosis in primary SjS by virtue of elevated levels of the B-cell survival factor, BAFF (B-cell activating factor) [7]. Another theory is that B cells in primary SjS accumulate and persist due to abnormal or inadequate regulation by other cells of the immune system. Borrowing an example from the field of tumor immunology, B cells that are transformed by Epstein-Barr virus are efficiently killed by T cells and natural killer cells [8]. T cells in SjS not only may be derelict in their duties to constrain or kill transformed B cells but may have joined forces with the enemy: SjS T cells are the predominant inflammatory cell population in the exocrine gland lesions, appear to respond to autoantigens on apoptotic cells, secrete pro-inflammatory cytokines, and stimulate B cells (reviewed in [2]).

Clonal expansions that are evident from abnormalities in the blood may reveal underlying processes that can evolve into a malignant B-cell neoplasia. For example, some patients with monoclonal gammopathy of uncertain significance (MGUS), a condition characterized by the presence of a monoclonal immunoglobulin protein present in the serum, can progress to multiple myeloma [9]. Similarly, a subset of patients with monoclonal B-cell lymphocytosis (MBL), a condition characterized by the presence of a clonal B-cell expansion in the peripheral blood, can progress to chronic lymphocytic leukemia [10].

We wondered whether a similar spectrum of conditions occurs in primary SjS, with some patients with primary SjS having polyclonal B-cell expansions, others having monoclonal expansions, and still others having transformed monoclonal expansions [4]. Since primary SjS is a systemic autoimmune disease, expanded B-cell clones in the circulation could both be pathogenic (autoreactive) and be linked to an increased risk of lymphoma. However, in primary SjS-associated lymphoma, the B-cell neoplasm is often of the mucosal associated lymphoid tissue (MALT) type [11]. It is not clear whether B-cell populations in the peripheral blood overlap with those in the glands and other tissues, although one intriguing report documents the presence of shared expanded clones in the lymph node tissue and peripheral blood of a patient with SjS who also had marginal zone lymphoma [12].

The results from several studies suggest that therapy with the B cell-depleting antibody, rituximab (anti-CD20), leads to symptomatic improvement in patients with primary SjS [13-15]. Furthermore, rituximab is known to modulate the antibody repertoire, particularly in antigen-experienced B cells [16,17]. We therefore reasoned that rituximab therapy might also alter the circulating repertoire of expanded B-cell clones in patients with primary SjS who did not have lymphoma or overt evidence of MGUS or MBL. If CD20 antibody depletion of B cells eliminated expanded B-cell clones and re-set the B-cell repertoire, this approach might favorably modify the disease course in primary SjS by eliminating pathogenic B-cell clones that could be contributing to autoimmunity or predisposing to lymphoma. Conversely, the finding of persistent B-cell clones despite CD20 B-cell depletion therapy with rituximab implies that the restoration of the B-cell repertoire to a pre-disease state is incomplete at best and that the underlying mechanisms responsible for driving the disease process remain unchecked by B-cell depletion. Furthermore, the analysis of diversification by somatic hypermutation of the antibody gene rearrangements within the expanded clone might reveal mechanistic insights into how the clone is being selected (either by self antigen or by negative regulation from the immune system). Therefore, we analyzed the B-cell repertoire of SjS subjects at baseline and after treatment with rituximab by single B-cell sorting and immunoglobulin heavy-chain gene sequencing to identify and molecularly characterize expanded B-cell clones in the blood.

## Methods

### Study subjects

Subjects with primary SjS, as defined by the revised American-European Consensus Group criteria, were enrolled in an open-label Autoimmunity Centers of Excellence-sponsored clinical trial of the B cell-depleting antibody, rituximab (anti-CD20). Clinical features of the study subjects have been described previously [15]. Subjects received two 1-gram doses of rituximab and 100 mg of methylprednisolone on days 0 and 15. For this study, the peripheral blood of six out of the 12 subjects with SjS were studied by flow cytometry, and antibody heavy-chain cloning was performed from single-sorted cells at baseline and at weeks 8, 14, 26, 36, and 52 after the second dose of rituximab [15]. Subjects provided informed consent to participate in this study, which was carried out in accordance with a study protocol that was approved by the institutional review boards of both the Perelman School of Medicine at the University of Pennsylvania and Duke University Medical Center.

### Single-cell cloning of immunoglobulin heavy-chain gene rearrangements

Memory and germinal center B-cell subsets (shown in Additional file 1: Figure S1) were recovered from the peripheral blood as described previously [18,19]. Memory and plasmablast (PB) B-cell subsets were sorted into single wells of 96-well polymerase chain reaction (PCR) plates, and rearranged immunoglobulin heavy-chain (IgH) genes were cloned and sequenced as described previously [19].

### Cloning of the germline *VH1-69* immunoglobulin heavy-chain gene rearrangement

Genomic DNA from subject 2 (SjS2) was subjected to PCR amplification with primers that flanked the germline *VH1-69* sequence. The primers used were *VH1-69* germline forward: 5′-GTG CCC TGA GAG CAT CAC ATA ACA-3′ and VH1-69 germline reverse: 5′-TTC TCC CTC AGG GTT TCT GAC ACT-3′. The cycling conditions were 10 minutes at 94°C, followed by 40 cycles of 94°C for 30 seconds, 58°C for 30 seconds, and 72°C for 30 seconds, followed by 20 minutes at 72°C. Amplicons were TA-cloned (TOPO TA kit; Life Technologies, Grand Island, NY, USA) and sequenced.

### Identification of VH, DH, and JH immunoglobulin heavy-chain gene sequences

For each sequenced immunoglobulin gene rearrangement, the most closely matching germline VH, DH, and JH alleles were identified, along with the CDR3 length, functional status (in frame, out of frame, termination codon), and predicted (translated) amino acid sequence by local alignment comparison with the ImMunoGeneTics (IMGT) database [20]. Alignments were verified by using the high-throughput sequencing analysis algorithms from IMGT (V-QUEST version 1.1.2 [21]), and individual sequences were spot-checked with IgBLAST (National Library of Medicine). To avoid confusing mutations with sequencing imperfections, we considered only positions 10 to 381 of the sequences (by IMGT numbering). The numbers of identifiable IgH variable, diversity and joining (VDJ) rearrangements for each subject at each time point are summarized in Additional file 2: Table S1.

### Clonal immunoglobulin gene tree construction

FASTA sequences for all of the clonal variant sequences were analyzed and graphically displayed by using neighbor joining with ClustalX, version 2.1 [22] and by using the default parameters.

### Detecting selection from immunoglobulin gene sequence mutation patterns

We used the focused selection test, which is explained in detail in [23], to detect the forces of selection in the complementarity-determining regions (CDRs) and in the framework regions (FWRs) of the B-cell clones. This test has been shown to be the only one to give reliable and accurate results inferring selection from replacement-to-silent (R/S) ratios [24] and takes into account micro-sequence specificity [25,26] and transition bias [27] in somatic hypermutation. Taking these two factors into account, we calculated for any given set of nucleotides their expected mutability (that is, their relative likelihood to mutate). To estimate the expected level of selection, we count the number of silent mutations in the entire sequence and then use a micro-sequence-based targeting model of somatic mutation as described in [28] and the actual sequence composition of the mutant’s source germline sequence to estimate the expected number of R mutations [29,30]. We then used the mutability of the sequence as a background to calculate, under a condition of no selection, the expected rate of R mutations in the area of interest (CDR or FWR) and the S mutations throughout the sequence. In this way, we avoided mixing potentially conflicting forces of selection in FWR and CDR. An overabundance of R mutations was considered an indication of positive selection, and a lack of R mutations was considered an indication of negative selection. When a set of clonally related sequences was analyzed, all unique mutations were grouped together, since mutations that occurred multiple times were probably an indication of common ancestry rather than being mutations that occurred more than once. In this manner, we raised the sensitivity above that obtained by analyzing a single sequence without spuriously counting mutations twice.

We further quantified selection strength by using BASELINe [30]. This algorithm extrapolates the distribution of potential models of selection pressures that could result in the observed patterns of mutation. To accomplish this task, BASELINe considers all mutations from the same condition and considers their origin (clone/sequence/CDR/FWR). In this way, we compared clones with unequal sequence numbers. BASELINe outputs a selection strength (positive or negative) whose certainty is expressed by the width of the distribution curve. All calculations presented here were done with BASELINe’s web version [31].

## Results

We have previously described the results of our open-label pilot trial of rituximab therapy for primary SjS [15]. In this companion study, our goal was to determine whether therapy with rituximab altered or re-set the repertoire in patients with primary SjS, using expanded B-cell clones as a read-out.

### B-cell clones are frequent in peripheral blood memory B cells and plasmablasts of rituximab-treated Sjögren’s syndrome subjects

To explore the effects of CD20 B-cell depletion on the B-cell repertoire, antibody heavy-chain gene rearrangements were cloned and sequenced from sorted PBs and memory B cells (see Methods and Additional file 1: Figure S1) of six subjects with SjS before and at various time points after therapy with rituximab. Since B-cell clonal expansions have previously been described in patients with primary SjS [32-34], we reasoned that it would be possible to find and track an expanded B-cell clone in one or more of the subjects over the different study time points.

Here we define clonally related heavy-chain sequences as those that share the same VH (variable), DH (diversity), and JH (joining) gene segments; have the same CDR3 (third complementarity-determining sequence) length; and have highly related CDR3 sequences (identical or differ by only one amino acid or up to three nucleotides). Among the estimated tens of billions of different CDR3 sequences [35], rearrangements that share the same VH, DH, and JH; have the same CDR3 length; and have highly similar CDR3 sequence overall are very likely to be derived from clonally related B cells. Because the sequences were recovered from single cells, finding the same sequence in two separate cells (which were separately amplified), even if they were obtained at the same time point, was considered to be indicative of clonal expansion.

**Figure 1:**
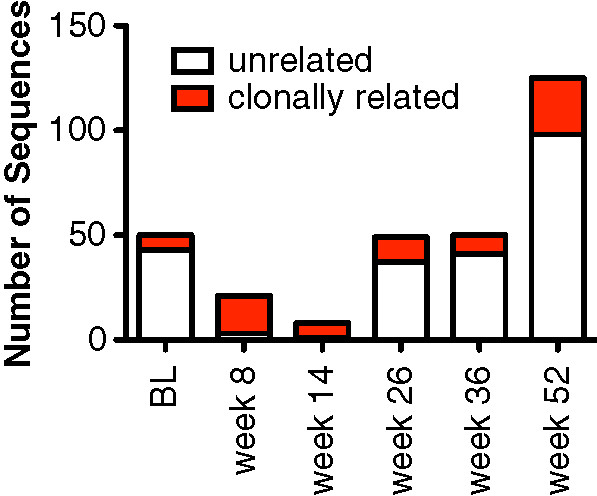
**Clonally related and unrelated sequences over time.** The stacked bar graph indicates the numbers of immunoglobulin heavy-chain (IgH) sequences with identifiable rearrangements that were obtained by single-cell sequencing in all six subjects with Sjögren’s syndrome (SjS). White bars indicate unrelated IgH rearrangements. Red bars indicate IgH rearrangements that are present in at least two independent sequences and are therefore deemed to be clonally related. The numbers of unique and clonally related sequences obtained for each subject at each time point are given in Additional file 2: Table S1. BL, baseline.

A remarkable feature of the antibody heavy-chain sequencing data is that clones were identified despite sequencing relatively few B cells: out of 303 sequences, 12 independently expanded clones were identified. Members of these clones comprised 80 out of the 303 sequences (26%). The distribution of clonally related sequences and the number of sequences sampled at each time point are shown in Figure 1 and in Additional file 2: Table S1. Owing to the low numbers of circulating B cells, very few sequences were obtained between weeks 8 and 26, but a high fraction of the sequences that were obtained at these time points (mostly from SjS2) were clonally related.

Analysis of the heavy-chain sequences revealed that the B-cell clones from different subjects (and in the same subjects) did not share any obvious sequence similarities (Table 1). Additional file 2: Table S1 summarizes the clonal expansions that were identified in the subjects with SjS. In SjS1, we identified two expanded clones among only 24 total sequences. In SjS3, we identified two clones in 30 sequences, and in SjS4 we identified two clones in 65 sequences. But the most interesting subject was SjS2, who had a large expanded clone that was present during all of the time points. Members of this clone were present in the circulation in spite of rituximab therapy and consisted of over 50 independently amplified sequences (FASTA files are provided in Additional file 3: Table S2). Also in SjS2, four clones (including the large clone) were present at baseline. These data indicate that clones are also detectable in the memory B-cell repertoire in at least some subjects prior to the administration of rituximab.

**Table 1:** CDR3 sequence features of expanded B-cell clones

**Clone**	**VH**	**DH**	**JH**	**CDR3 (amino acid)**
1-1	VH3-30-03*01	D3-3*01	JH6*03	CASPYYDFWSGYYMDYYYYYMDVW
1-2	VH3-30*03	DH6-19*01	JH5*01	CAKEAGSSGRAGWFDPW
2-1	VH1-69*01	DH4-23*01, DH4*02	JH4*02	CARGTGDHTTVVTPFDYW
2-2	VH3-23*04	DH6-13*01	JH3*01	CAKAVAPVGSAYDVW
2-3	VH1-2*04	DH3-10*01	JH3*02	CARDSSGGGNDAFDMG
2-4	VH4-59*01	DH3-22*01	JH6*02	CARGMKVVAGYYYYGMDVW
2-5	VH3-23*04	DH6-19*01	JH3*02	CAKAAAVGSAYDIW
2-6	VH4-59*01	DH4-17*01	JH2*01	CAREDYGDYVRW
3-1	VH4-31*03	DH3-10*01	JH6*02	CAREGNTFIRGVIGWDPKPMDVW
3-2	VH1-46*01	DH3-10*01	JH4*02	CARDGSHYDFDYW
4-1	VH3-30*02	DH1-1*01	JH6*03	CARDSRGATGTSYYYYYMDVW
4-2	VH1-2*02	DH6-19*01	JH4*02	CARDAGSAGNYDTAVAGGGFVDYW

Shown are the best matches for the corresponding VH, DH, and JH gene alleles and the translated amino acid junction (CDR3) based upon the ImMunoGeneTics (IMGT) server output for all of the expanded B-cell clones. The clone numbers indicate the subject number followed by a unique identifier for the clone. For example, 2-1 refers to first (and largest) expanded clone in Sjögren’s syndrome subject 2 (SjS2). Expanded clones are molecularly defined as described in the text. DH, diversity gene segment; JH, joining gene segment; VH, variable gene segment.

The large clone from SjS2 was identified as an in-frame rearrangement of VH1-69, DH7-27, DH4-23, and JH4-02. Additional file 4: Figure S2 shows the alignment of the most frequently recovered member of the clone with the corresponding germline VH, DH, and JH sequences. We could not account for 10 nucleotides in the CDR3 by their presence in the germline VH, DH, and JH genes. Since these 10 nucleotides were shared in nearly all of the sequences, we inferred that these nucleotides most likely arose by junctional diversification (n- or p-addition) rather than by somatic hypermutation.

### Members of the large expanded clone are found in circulating plasmablast and memory B-cell pools

**Figure 2:**
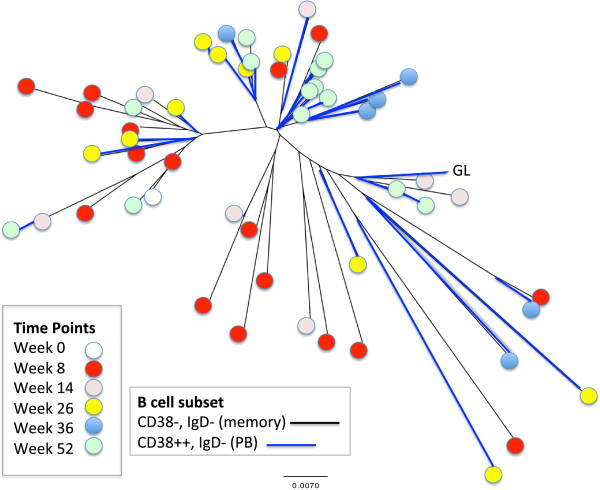
**Time point and B-cell subset distribution of the large expanded clone.** The graphical ClustalX alignment of all of the expanded clone members from Sjögren’s syndrome subject 2 (SjS2) is based upon their degree of nucleotide sequence similarity. The circles at the end of each branch indicate the time point, and the colors of the branches show the memory B-cell subset from which the clone member was recovered. Blue indicates CD38^++^ (plasmablasts, PB), and black indicates CD38^−^ (memory cells). GL, inferred germline sequence.

The distribution of the members of this large expanded clone with respect to time point and B-cell subset is shown graphically, using neighbor joining in Figure 2. The lengths of the branches are fairly long (distant) when compared with the germline (GL) sequence, consistent with mutations in most of the clone members. The shape of the clone alignment is more like a “bush” than a “tree”, showing no clear single direction of mutation. Rather, there are several small branches with sequence variants. The sequence variants do not display a clear-cut progression with time, at least during the time points that clonal variants were surveyed. This suggests that the major changes in the clone sequence likely occurred prior to this analysis of the clone. Of note, members of the clone were present in both CD38^++^ (PB) and CD38^+/−^ class-switched (IgD^−^) memory (CD27^+^) B cells, indicating potential clonal overlap between these two pools of CD27^+^ peripheral B-cell pools. Small clusters of sequence variants that coincide with the memory B-cell subset can be seen, further suggesting that selection for the clone members could be distinctive within the PB and memory B-cell subsets.

### Somatic mutations are frequent in the large expanded clone

To gain further insight into how the expanded clone was selected, we analyzed its pattern of mutation. Although it is possible that some of the intra-clonal variation is due to PCR error, we do not think that PCR error contributes substantially to the sequence diversity of this clone based on the large number of shared mutations between sequences and the ability to independently amplify identical sequences from different B cells.

We also considered the possibility that some of the mutations represented variations in the GL sequence due to the hypothetical presence of an unusual *VH1-69* allele in subject SjS2. We therefore cloned the subject’s GL VH1-69 gene by using primers that were present in intronic sequences surrounding the unrearranged *VH1-69* gene (see Methods). We obtained only a single *VH1-69* sequence out of several independent PCRs (data not shown). Upon alignment (see Methods), the VH sequence matched VH1-69*01 (100% identity, Additional file 4: Figure S2). We therefore conclude that SjS2 is most likely homozygous for the VH1-69*01 allele and that the majority of the sequence differences among different members of the expanded clone are due to somatic hypermutation. Additional file 5: Table S3 summarizes the unique somatic mutations in the members of the expanded clone, relative to the GL sequences for VH1-69*01, JH4-02, and the CDR3 (comprised of DH7-27, DH4-23, and consensus in the areas with presumed n and p nucleotide insertions at the junctions).

### Many of the somatic hypermutations in the expanded clone are silent

**Figure 3:**
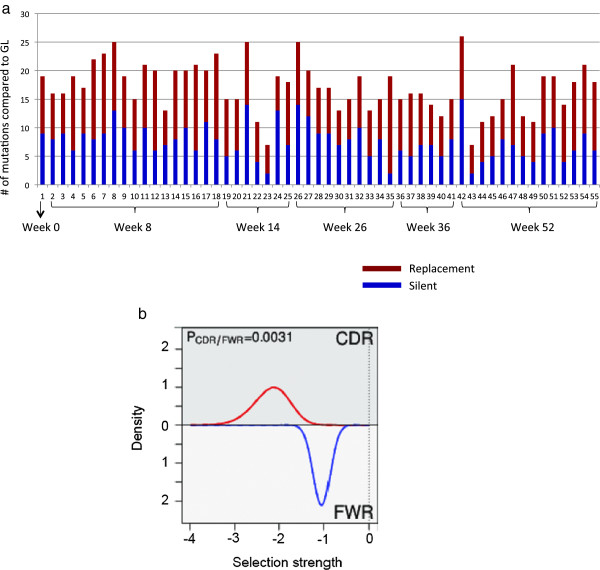
**Evidence for negative selection in the large expanded clone. (a)** High frequency of silent mutations in members of the large expanded clone. Each sequence variant (member) of the clone was compared with the germline sequence to determine the numbers and kinds of mutations it contained. The heights of the bars represent the number of mutations, and the proportions of the bars that correspond to replacement (R, red) or silent (S, blue) mutations are shown. Sequence variant numbers are given on the x-axis, as are their corresponding time points. Nucleotide positions 10-381 (ImMunoGeneTics, or IMGT) were used for the alignment. **(b)** Graphical output of BASELINe analysis [30] (see Methods). Negative values indicate negative selection, and positive values indicate positive selection. Confidence in the magnitude of selection is depicted by the width of the curve; the narrower the curve, the more confident we are of the results. Both the complementarity-determining region (CDR) and the framework region (FWR) show strong negative selection. The CDR shows evidence of significantly more negative selection than the FWR (*P* ≤0.0031).

When the nucleotide sequences of the clone members are compared with the corresponding GL sequence, approximately half of the mutations, on average, are silent (Figure 3a). If the clone were positively selected, one would instead expect to find an increased frequency of R mutations compared with S mutations. A high frequency of S mutations is also not what one would expect to find at random. Owing to redundancies in the genetic code, the R/S ratio without any selection is generally approximately 3 to 1 in favor of R mutations. In contrast, the trends in this clone are all in the opposite direction: we find an overabundance of S mutations. However, the analysis in Figure 3a ignores the fact that many of the mutations among different members of the clone are shared and therefore are unlikely to be independent events. Therefore, we performed a more rigorous computational analysis of the somatic hypermutation pattern that took the shared mutations as well as their locations in the V region into account.

Not surprisingly, the computational analysis of the somatic hypermutation pattern was also consistent with negative selection in both the CDRs and the FWRs. Using the focused test of selection, replacement mutations are significantly under-represented among the mutations that were identified (*P* <0.05 at baseline and *P* <0.001 at other time points and overall) in all regions and overall (Table 2). To identify more clearly the strength of the negative selection and not just its presence, we used BASELINe on members of the clone (Methods), and we see quite clearly that there are high levels of negative selection (Figure 3b). Thus, there is negative selection in both the CDRs and the FWRs of the members of this clone. Furthermore, the other expanded clones identified in SjS2 and some of the other subjects exhibit somatic mutations that are consistent with negative selection (Additional file 6: Table S4). Thus, the mutation pattern found in the big clone in SjS2 is not unique to this particular clone and may be a more general feature of expanded clones in SjS.

**Table 2:** Observed and expected numbers of unique R and S mutations per time point and overall in the large clone of Sjögren’s syndrome subject 2

**Time**	**N**	**CDR R obs**	**CDR S obs**	**FWR R obs**	**FWR S obs**	**CDR R exp**	**CDR S exp**	**FWR R exp**	**FWR S exp**
All	55	6	24	46	47	27.18	9.59	62.36	23.86
Week 0	1	1	1	6	9	3.76	1.33	8.59	3.32
Week 8	17	5	18	30	36	19.67	6.94	45.12	17.27
Week 14	7	1	11	17	22	11.27	3.98	25.86	9.89
Week 26	10	1	14	22	25	13.70	4.84	31.43	12.03
Week 36	6	2	5	18	22	10.39	3.67	23.83	9.12
Week 52	14	1	5	10	22	8.40	2.96	19.27	7.37

Shown are the observed (obs) and expected (exp) numbers of replacement (R) and silent (S) mutations within each unique clonally related variant at each time point for members of the big clone in Sjögren’s syndrome subject 2. Both the complementarity-determining region (CDR) and the framework region (FWR) exhibit significant negative selection at all time points and overall (*P* <0.05 by the focused selection test; see Methods). N, number.

**Figure 4:**
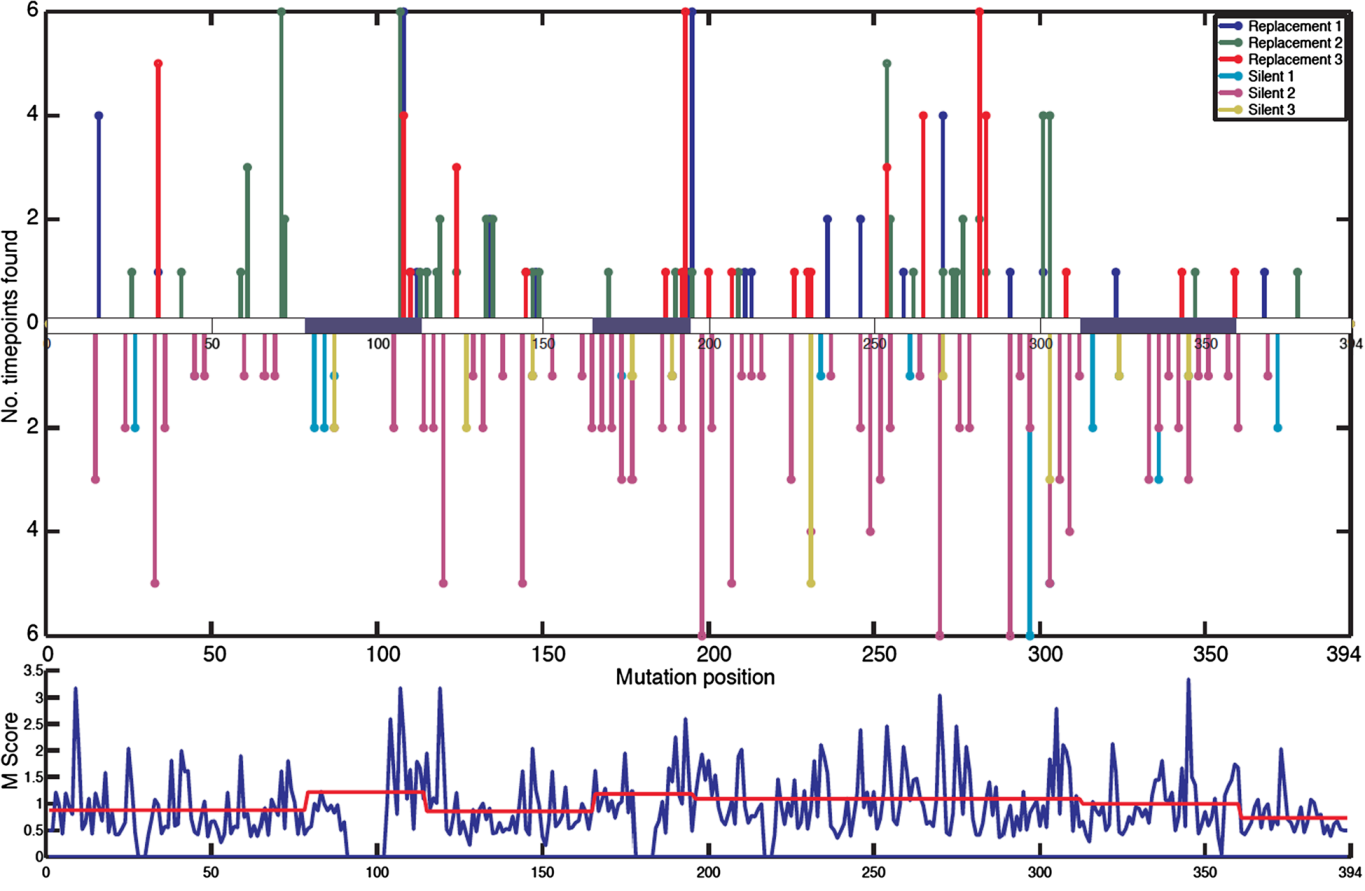
**Time points that include specific mutations relative to the imputed germline sequence.** (Top) number of time points with specific R mutations (line going up) and S mutations (line going down) at a specific position in the V region sequence. Some positions allow multiple different nucleotide substitutions. For this reason, each R or S mutation can be a combination of up to three lines, each of which represents a different nucleotide substitution at that position. The complementarity-determining regions (CDRs) are marked in blue shaded boxes. (Bottom) mutability score (see Methods) by position. A mutability score above 1 indicates a relatively increased tendency to mutate. The average mutability per region—CDR or framework region (FWR)—is indicated by the red line.

Two additional features of this large B-cell clonal expansion become apparent when examining the distribution of mutations of the clonal variants at the six different time points (Figures 2 and 4 and Table 2). First, there is very little detectable accumulation of mutations over time, as most mutations are already observed at week 8. Second, the common mutations (that is, those found in many sequences across all time points; Figure 4) are not enriched for R mutations when compared with the rare mutations (that is, those found in only a few sequences and in one time point). We therefore considered it possible that none of these mutations is important for the formation of this lineage (that is, they may not confer selective advantage compared with the non-mutants). The common mutations could simply be derived from the same lineage that was present in the subject prior to the study.

## Discussion

Clonal expansion is a fundamental property of B cells that participate in an adaptive immune response. In patients with SjS, the identification, characterization, and tracking of expanded B-cell clones over time can provide insights into how clones are selected and whether they are affected by therapy. In this study, we show that expanded B-cell clones are present in the peripheral blood of patients with primary SjS undergoing B-cell depletion therapy with rituximab. There were three main findings: (1) a large expanded clone persisted in the blood of a patient with primary SjS (subject SjS2) despite B-cell depletion with rituximab; this finding indicates that rituximab does not fully re-set the B-cell repertoire in at least some patients with primary SjS; (2) the relative proportion of expanded B-cell clones among sequences recovered from memory and PB cells was highest during the period of maximal B-cell lymphopenia; this finding suggests that the sensitivity for detecting clonal expansions is greatest during periods of iatrogenic or disease-induced lymphopenia; (3) the expanded clones harbored numerous silent mutations; this finding indicates that expanded clones are under negative selection. The frequency and kinds of somatic mutations have potential implications for monitoring the longevity and pathogenic potential of expanded clones in patients with SjS.

### Expanded clones are readily detected following rituximab therapy

The presence of expanded B-cell clones and their persistence after rituximab therapy are consistent with the recently reported finding of large persistent B-cell clones in the glands of SjS patients treated with rituximab [36] and extend the concept of clonal persistence in SjS to the peripheral blood. The expanded B-cell clone in SjS2 persists over the entire 1-year period of the study. It is worth noting that the large clone in SjS2 comprised the highest fraction of the circulating memory B cells during the period of maximal B-cell lymphopenia. This apparent enrichment for the clone during lymphopenia could indicate resistance to rituximab. It is possible that B cells in SjS are intrinsically more resistant to depletion. To this point, autoimmune mice are more resistant to anti-CD20 depletion than wild-type mice [37], but the exact mechanism is unknown. Memory B cells and PBs (the latter expressing lower levels of CD20 and dominating during the B-cell nadir) may be more resistant to anti-CD20 depletion (reviewed in [38]). Yet another non-mutually exclusive possibility is that rituximab-resistant B cells reside in protective niches in the secondary lymphoid organs or the exocrine glands and are released with time from these sites during depletion [37,39]. Another possibility is that the expanded clone undergoes more exuberant homeostatic proliferation during the period of B-cell lymphopenia than polyclonal B cells. The B-lymphopenic state brought about by rituximab therapy is accompanied by increased levels of the B-cell survival factor, BAFF [40], which, in turn, could relax selection stringency and potentially allow self-reactive or multi-reactive B-cell clones to flourish. In primary SjS, this speculation is particularly apt, as BAFF levels are known to be elevated [41] and to increase after rituximab therapy in this disease [42,43], including the current subject cohort and in SjS2 in particular [15]. Furthermore, high BAFF levels appear to correlate with B-cell clonal expansion in the salivary glands and are associated with an increased risk of lymphoproliferative disorders [32].

Irrespective of the mechanism of clonal resistance, the facile detection of expanded B-cell clones after rituximab therapy points to a possible way to evaluate the risk of lymphoma or the effects of B-cell targeted therapy in patients with primary SjS. It may be easiest to identify expanded clones during the period of B-cell lymphopenia, when there may be a greater opportunity for oligoclonal expansions to be recognized. Selectively surveying the memory B-cell compartment also may have increased the sensitivity of detection of expanded clones, as expanded clones are readily recovered from circulating PBs in vaccinated individuals with intact immune systems [44].

### Molecular features of the expanded clone immunoglobulin heavy-chain rearrangements

The expanded clones recovered from the different subjects with SjS used a variety of VH genes. However, the big clone from SjS2 was interesting because it expressed a VH1-69/JH4 rearrangement with a D-D fusion that could be relevant to the loss of tolerance and disease pathogenesis. The *VH1-69* heavy chain includes hydrophobic amino acids in CDR2 that can adopt an unusual conformation and bind a variety of antigens [45,46]. *VH1-69* is frequently used in polyreactive and crossreactive antibody responses to HIV [47] and in the antibody responses to influenza [46] and hepatitis C virus (HCV) [48]. Moreover, molecular analysis of antibody gene rearrangements in salivary gland biopsies from patients with SjS reveals increased usage of *VH1-69*, and even specific H + L chain combinations (VH1-69 + Vκ3-20), that have also been recovered from HCV-associated non-Hodgkin lymphoma cases [49,50]. IgM+κ+B cells with rheumatoid factor activity are expanded in blood from patients with HCV-associated cryoglobulinemia that express VH1-69/JH4 and Vκ3-20 [51], suggestive of a common antigenic drive. Vκ3-20 has also been recovered repeatedly in anti-Ro 60 antibodies from separate individual patients with SjS [52]. Of note, SjS2 did have serologic evidence of rheumatoid factor (data not shown) but was HCV-negative and did not have clinical evidence of lymphoma at the time of the rituximab trial.

The D-D fusion found in the SjS2 big clone is fairly uncommon in the normal antibody repertoire, reportedly occurring in approximately 1/800 rearrangements from the blood B cells of healthy individuals [53]); however, D-D fusions may be more common in autoimmune strains of mice [54]. D-D fusions tend to lengthen the CDR3, although this particular CDR3 sequence is not exceptionally long. The translated CDR3 sequence, which for most members is CARGTGDHTTVVTPFDYW, is highly conserved among the sequence variants of this expanded clone. This sequence, like many other CDR3 sequences, has several hydrophilic residues at the VH end and more hydrophobic residues on the JH end. Whether such a mildly amphipathic sequence with extra length afforded by the second D gene segment can confer greater multireactivity is unclear and may depend critically upon the light chain, which is a major determinant of rheumatoid factor activity [55,56].

### Silent mutations in the expanded clones

The most striking feature is the overall predominance of silent mutations in the heavy-chain sequences among members of the expanded clone, best exemplified in the big clone of subject SjS2. This pattern of mutations is indicative of strong negative selection and may occur because the clone has evolved to the point where it can no longer improve its affinity through mutation; any further replacement mutation thereby might lower the antibody affinity for its antigen. It seems quite clear that whatever characteristic is being ‘protected’ by the negative selection is related to antigen binding given that the negative selection is more pronounced in the CDRs than in the FWRs (Figure 3b, *P* ≤0.0031). As such, the clone could be highly dependent on a specific antigen. A second possibility is that it is not selection for binding to one antigen but selection for binding to several different antigens that results in a survival advantage. In this scenario, mutations of the CDRs would limit the receptor’s potential interactors, by making it less multireactive. It is intriguing in this respect that some of the commonly encountered autoantigens in systemic autoimmune disease, including the Ro and La antigens that are commonly targeted in SjS, appear to have high degrees of molecular disorder or entropy [57]. Antibody binding to a high-entropy molecule would require a greater activation energy than binding to a lower-entropy molecule and may thereby introduce structural or functional constraints in either the FWR or CDRs. Of course, if the antibody were a rheumatoid factor, it would be able to interact via a wide range of antigens by forming complexes with antibodies bound to other antigens. A third possibility is that the antibody is being selected for some property other than antigen binding, such as the ability to multimerize or self-associate [58].

In a B-cell that has lost negative regulatory signals from tonic antigen receptor crosslinking, chronic autoantibody crosslinking via persistent self or foreign antigen (model 1), multireactivity (model 2) or antibody self-association (model 3) could promote survival. It is also possible that B cells expressing certain variants of the antibody are kept in check by the immune system. A more detailed analysis of expanded clones and their functional characterization will be needed to distinguish between these intriguing potential modes of negative selection. The existence of negative selection in the CDR serves to remind us that the divisions of CDR and FWR and the impact of mutation are not set in stone. At times, a beneficial mutation may improve antibody affinity by changing an amino acid in the FWR and another mutation may degrade affinity and receptor function by changing an amino acid in the CDR. More generally, high affinity and receptor specificity are not in and of themselves always the goals of somatic selection. As in larger-scale processes of selection that underlie evolution, what determines the function under selection is the specific environment and its constraints.

## Conclusions

This study documents the presence of expanded B-cell clones among memory cells and PBs in the circulation of subjects with SjS being treated with rituximab. Expanded clones were readily detected among circulating memory and PB B-cell subsets during periods of B-cell lymphopenia. One SjS subject had an expanded clone that was present prior to and at various times after B-cell depletion therapy. A detailed analysis of the nucleotide sequences of 55 members of this clone revealed a high proportion of silent mutations, suggesting that the clone was under chronic negative selection. Some of the other expanded clones isolated from other rituximab-treated SjS subjects also had frequent silent mutations. The frequency of silent mutations in an expanded clone and its B-cell subset distribution may provide a means of measuring the chronicity and selection of potentially pathogenic B cells in humans.

## Abbreviations

BAFF: B-cell activating factor; CDR: complementarity-determining region; DH: diversity gene segment; FWR: framework region; GL: germline; HCV: hepatitis C virus; IgH: immunoglobulin heavy-chain; IMGT: ImMunoGeneTics; JH: joining gene segment; MBL: monoclonal B-cell lymphocytosis; MGUS: monoclonal gammopathy of uncertain significance; PB: plasmablast; PCR: polymerase chain reaction; SjS: Sjögren’s syndrome; VH: variable gene segment.

## Competing interests

ML and EWS received funding from Genentech (South San Francisco, CA, USA) for mechanistic assays that accompanied the original clinical trial [15], but Genentech did not fund the studies in this report. ML currently serves as a consultant for Genentech and has research funding from Genentech. The other authors declare that they have no competing interests.

## Authors’ contributions

ML contributed to the conception and design of the study. ELP contributed to the conception and design of the study and helped to analyze the sequence data and to draft the manuscript. PC and EWS contributed to the conception and design of the study and helped to analyze the clinical data. PM and LL helped to perform the cell sorting and sequencing experiments. ML helped to perform the cell sorting and sequencing experiments and to draft the manuscript. UH helped to analyze the sequence data and to draft the manuscript. WM, BZ, and NH helped to analyze the sequence data. All authors read and approved the final manuscript.

## Supplementary Material

Additional file 1: Figure S1 Overview of sorting and single-cell polymerase chain reaction (PCR) workflow. Peripheral blood mononuclear cells were stained with antibodies to CD3, CD14, CD16, CD19, IgD, and CD38. CD19^+^, CD3^−^, CD14^−^, and CD16^−^ lymphocytes were analyzed for IgD and CD38 expression, and memory cells (CD38^+^, IgD^−^) and plasmablast (PB) phenotype cells (CD38^++^, IgD^−^) were sorted into 96-well plates for single-cell amplification and cloning as described in Methods. Click here for file

Additional file 2: Table S1 Numbers of sequences and clonally related sequences from the six subjects with Sjögren’s syndrome (SjS). Shown are the six subjects (SjS1 to SjS6) and the number of sequences with identifiable (VDJ) rearrangements recovered from plasmablast (PB) or memory B cells at each time point. Also shown are the numbers of sequences that are members of the 12 expanded clones (defined as sequences that share the same VH, DH, and JH and have a very similar CDR3 sequence, within one amino acid and within three nucleotides) and the percentage of total sequences that are members of expanded clones (these data are graphically shown in Figure 1). The final column lists each clone and the number of times (in parenthesis) that the clone was identified in each subject. For example, in SjS2, one clone was found in all six time points, indicated as 1(6), one clone was found at two time points, indicated as 1(2), and four clones at one time point, 4(1). BL, baseline; DH, diversity gene segment; JH, joining gene segment; VH, variable gene segment. Click here for file

Additional file 3: Table S2 List of clonally related sequences of the large expanded clone from Sjögren’s syndrome subject 2 (SjS2) that were analyzed for their mutation pattern. Shown are the sequences (in FASTA format) and their corresponding time points. Sequence names include a unique identifier that details the CD38 status of the B-cell subset from which the sequence was cloned (CDR38^++^ for plasmablasts or CD38^+/−^ for memory cells). Click here for file

Additional file 4: Figure S2 Alignment of the most common clone sequence with germline sequences. Shown is an alignment of the most common clone sequence (upper case) to the most closely matching germline sequences in the ImMunoGeneTics (IMGT) database (lower case). Probable regions of junctional diversification are indicated (n for n-addition and p for p-addition), although somatic hypermutations in the CDR3 sequence cannot be ruled out, since the germline version of the CDR3 sequence is not available for comparison. Click here for file

Additional file 5: Table S3 List of unique mutations in the large expanded clone from Sjögren’s syndrome subject 2 (SjS2). List of unique mutations found in the 55 sequences in Table S2, organized by position. For each mutation, we list the germline codon it mutated from, its position, the mutant codon the mutated nucleotide is part of, whether that change by itself would be an S or an R mutation, and the number of sequences in which it is found. Note that some codons are mutated in more than one position and so will appear more than once. Click here for file

Additional file 6: Table S4 Analysis of somatic hypermutation selection in other expanded clones. Following the same format as Table 2, this table summarizes the selection in all of the clones identified in our experiments. Overall they exhibit negative selection, much like the large VH1-69 clone in Sjögren’s syndrome subject 2 (SjS2). However, their small numbers weaken the strength of detection. Click here for file
