# Supplementary figures and images for: Persistence and selection of an expanded B-cell clone in the setting of rituximab therapy for Sjögren’s syndrome
Source: Arthritis Res Ther. 2014 Feb 11;16(1):R51. doi: 10.1186/ar4481 (PMC3978607; doi:10.1186/ar4481)

Fig. S1: Overview of sorting and single cell PCR workflow

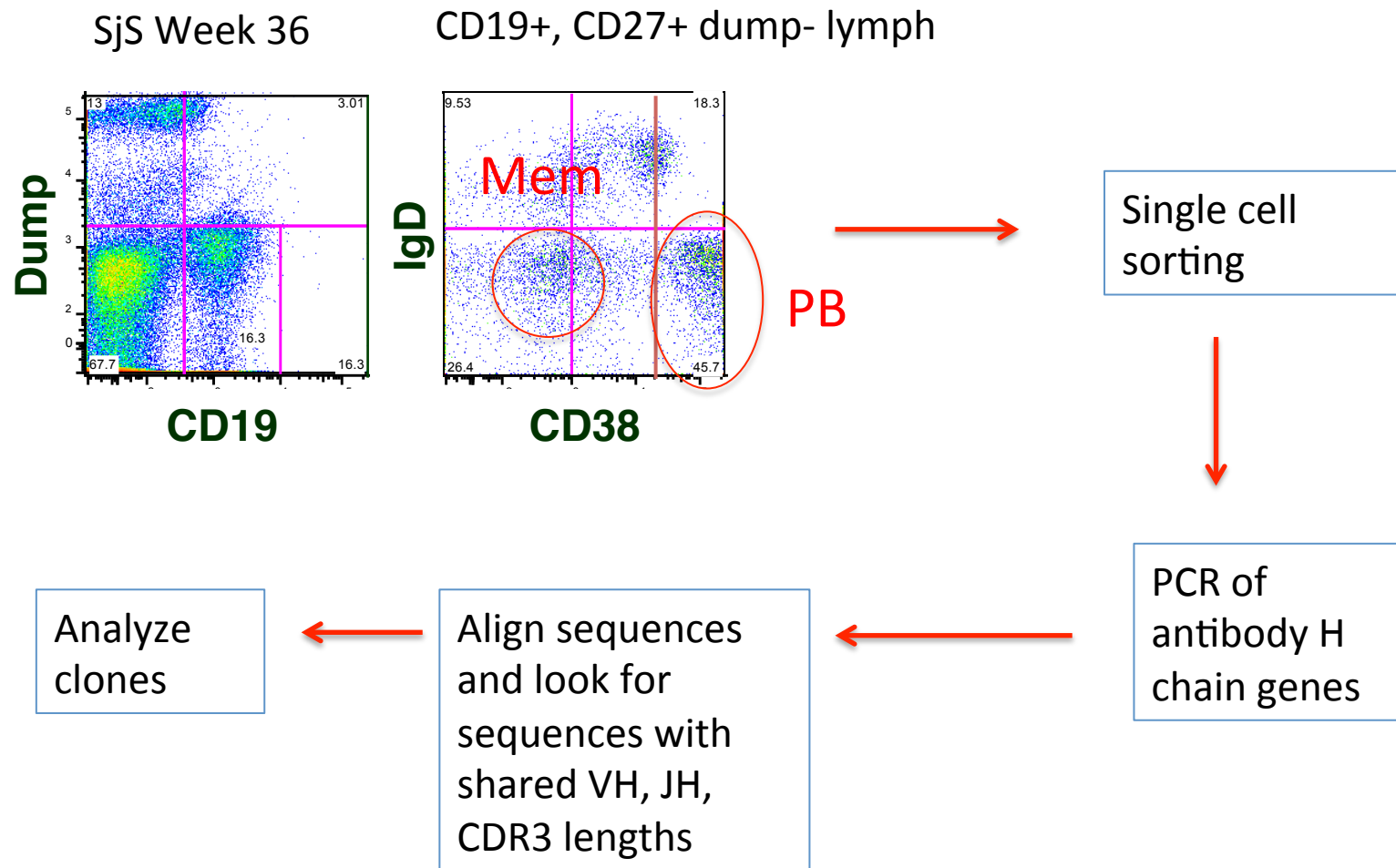

Supplement: Additional file 1: Figure S1 — Overview of sorting and single-cell polymerase chain reaction (PCR) workflow. Peripheral blood mononuclear cells were stained with antibodies to CD3, CD14, CD16, CD19, IgD, and CD38. CD19+, CD3−, CD14−, and CD16− lymphocytes were analyzed for IgD and CD38 expression, and memory cells (CD38+, IgD−) and plasmablast (PB) phenotype cells (CD38++, IgD−) were sorted into 96-well plates for single-cell amplification and cloning as described in Methods. [file ar4481-S1.pdf]
