# Supplementary material for: Persistence and selection of an expanded B-cell clone in the setting of rituximab therapy for Sjögren’s syndrome
Source: Arthritis Res Ther. 2014 Feb 11;16(1):R51. doi: 10.1186/ar4481 (PMC3978607; doi:10.1186/ar4481)
Supplement: Additional file 2: Table S1 — Numbers of sequences and clonally related sequences from the six subjects with Sjögren’s syndrome (SjS). Shown are the six subjects (SjS1 to SjS6) and the number of sequences with identifiable (VDJ) rearrangements recovered from plasmablast (PB) or memory B cells at each time point. Also shown are the numbers of sequences that are members of the 12 expanded clones (defined as sequences that share the same VH, DH, and JH and have a very similar CDR3 sequence, within one amino acid and within three nucleotides) and the percentage of total sequences that are members of expanded clones (these data are graphically shown in Figure 1). The final column lists each clone and the number of times (in parenthesis) that the clone was identified in each subject. For example, in SjS2, one clone was found in all six time points, indicated as 1(6), one clone was found at two time points, indicated as 1(2), and four clones at one time point, 4(1). BL, baseline; DH, diversity gene segment; JH, joining gene segment; VH, variable gene segment. [file ar4481-S2.pdf]

Table S1

Subject	BL	week 8	week 14	week 26	week 36	week 52	total	clones (time points)
SjS1					2	22	24	2 (1)
SjS2	13	21	8	45	26	64	177	1 (6), 1 (2), 4 (1)
SjS3	4			4	13	9	30	2 (1)
SjS4	26				9	30	65	1 (2), 1 (1)
SjS5	2						2	None
SjS6	5						5	None
total sequences	50	21	8	49	50	125	303	
clonal sequences	7	18	7	12	9	27	80	
percent clonal	14	86	88	24	18	22	26	
