# Supplementary material for: Persistence and selection of an expanded B-cell clone in the setting of rituximab therapy for Sjögren’s syndrome
Source: Arthritis Res Ther. 2014 Feb 11;16(1):R51. doi: 10.1186/ar4481 (PMC3978607; doi:10.1186/ar4481)
Supplement: Additional file 3: Table S2 — List of clonally related sequences of the large expanded clone from Sjögren’s syndrome subject 2 (SjS2) that were analyzed for their mutation pattern. Shown are the sequences (in FASTA format) and their corresponding time points. Sequence names include a unique identifier that details the CD38 status of the B-cell subset from which the sequence was cloned (CDR38++ for plasmablasts or CD38+/− for memory cells). [file ar4481-S3.pdf]

Table S2

>>Germline
CAGGTGCAGCTGGTGCAGTCTGGGGCT---
GAGGTGAAGAAGCCTGGGTCCTCGGTGAAGGTCTCCTGCAAGGCTT
CTGGAGGCACCTTC------------
AGCAGCTATGCTATCAGCTGGGTGCGACAGGCCCCTGGACAAGGGCTTGA
GTGGATGGGAGGGATCATCCCTATC------TTTGGTACAGCAAACTACGCACAGAAGTTCCAG---
GGCAGAGTC
ACGATTACCGCGGACGAATCCACGAGCACAGCCTACATGGAGCTGAGCAGCCTGAGATCTGAGGACACGGCCGTG
T
ATTACTGTGCGAGAGGAACTGGGGACCACACTACGGTGGTAACTCCTTTCGACTACTGGGGCCAGGGAACCCTGG
T
C
">38++/IGD-/27+|G4-VH1,3|Wk0"
NNGGTGCAGCTGGTGCAGTCTGGGGCT---
GAACTGAAGAAGCCTGGGTCCTCGGTGAAGGTCTCCTGCAGGGCTT
CTGGAGGCACCTTC------------
AGCAAGTATGCTATCAGCTGGGTGCGACAGGCCCCTGGACAAGGGCTTGA
GTGGATGGGAGGGATCATTCCTATC------TTTGGTACACCCAATTACGCACAGAAGTTCCAG---
GGCAGAGTC
ACCATTACCGCGGACGAATCCACGACCACAGCCTACTTGGAACTGAGCAGCCTCAGATCTGAAGACACTGCCGTC
T
ATTACTGTGCGAGAGGAACTGGGGACCACACTACGGTGGTAACTCCTTTCGACTACTGGGGCCAGGGCACCCTGG
T
C
>38-/D-/27+|E5-VH1/3|Wk08
GAGGTGCAGCTGGTGCAGTCTGGGGCT---
GAACTGAAGAAGCCTGGGTCCTCGGTGAAGGTCTCCTGCAGGGCTT
CTGGAGGCACCTTC------------
AGCAAATATGCTATCAGCTGGGTGCGACAGGCCCCTGGACAAGGGCTTGA
GTGGATGGGAGGGATCATCCCTATC------TTTGGTACACCCAATTACGCACAGAAGTTCCAG---
GGCAGAGTC
ACCATTACCGCGGACGAATCCACGAGCACAGCCTACTTGGAACTGAGCAGCCTCAGATCTGAAGACACTGCCGTA
T
ATTACTGTGCGAGAGGAACTGGGGACCACACTACGGTGGTAACTCCTTTCGACTACTGGGGCCAGGGAACCCTGG
T
C
>38-/D-/27+|E6-VH1/3|Wk08
GAGGTGCAGCTGGTGCAGTCTGGGGCT---
GAACTGAAGAAGCCTGGGTCCTCGGTGAAGGTCTCCTGCAGGGCTT
CTGGAGGCACCTTC------------
AGCAAGTATGCTATCAGCTGGGTGCGACAGGCCCCTGGACAAGGGCTTGA
GTGGATGGGAGGGATCATCCCTATC------TTTGGTACACCCAATTATGCACAGAAGTTCCAG---
GGCAGAGTC
ACCATTACCGCGGACGAATCCACGAGCACAGCCTACATGGAACTGAGCAGCCTCAGATCTGAAGACACTGCCGTA

T
ATTACTGTGCGAGAGGAACTGGGGACCACACTACAGTAGTAACTCCTTTCGACTACTGGGGCCAGGGNNNNNNNN
N
N
>38-/D-/27+|E7-VH1/3|Wk08
NNGGTGCAGCTGGTGCAGTCTGGGGCT---
GAGTTGAAGAAGCCTGGGTCCTCGGTGAAGGTCTCCTGTAGGGCTT
CTGGAGGCACCTTC------------
AGTAAGTATGCTATCAGCTGGGTGCGACAGGCCCCTGGACAACGGCTTGA
GTGGATGGGAGGGATCATCCCTATC------TTTGGTACACCAAATTACGCACAGAAGTTCCAG---
GGCAGAGTC
ACAATTACCGCGGACGAATCCACGACTACAGCCTACATGGAACTGAGCAGCCTGACATCTGAGGATACGGCCATA
T
ATTACTGTGCGAGAGGAACGGGGGACCACACCACGGTGGTAACTCCTTTCGACTACTGGGGCCAGGGAACCCTGG
T
C
>38-/D-/27+|E8-VH1/3|Wk08
GAGGTGCAGCTGGTGCAGTCTGGGGCT---
GAGGTGAAGAAGCCTGGGTCCTCGGTGAAGGTCTCCTGCAGGGCTT
CTGGAGGCACCTTC------------
AGCAAATATGCTATCAGCTGGGTGCGACAGGCCCCTGGACAAGGACCTGA
GTGGATGGGAGGGATCATCCCTATA------TTTGGTACACCCAATTACGCACAGAAGTTCCAG---
GGCAGAGTC
ACGATTACCGCGGACGAATCCACGAGCACAGCCTACATGGAACTGAGCAGCCTCACATCTGAGGACACTGCCGTA
T
ACTATTGTGCGAGAGGAACTGGGGACCACACTACGGTGGTAACTCCCTTCGACTACTGGGGCCAGGGAACCCTGG
T
C
>38-/D-/27+|E10-VH1/5|Wk08
NNNNNNCAGCTGGTGCAGTCTGGGGCT---
GAGGTAAAGAAGCCTGGGTCCTCGGTGAAGGTCTCCTGCAAGGCTT
CTGGAGGCACCTTC------------
AGCAAATATGCTATCAACTGGGTGCGACAAAGTCCTGGACAAGGGCTTGA
GTGGATGGGAGGGATCATCCCTATC------TTTGGTACACCAAACTACGCACAAAAGTTCCAG---
GGCAGAGTC
ACGATTACCGCGGACGAATCTACGAGTACAGCCTACATGGAGGTGAGCAGTCTGAGATCTGAAGACACGGCCGTT
T
ATTACTGTGCGAGAGGAACTGGGGACCACACAACGGTGGTAACTCCTTTCGACTACTGGGGCCAGGGAACCCTGG
T
C
>38-/D-/27+|F2-VH5|Wk08
NNNNNNNAGCTGGTGCAGTCTGGAGCA---
GAGGTGAAGAAGCCTGGGTCCTCGGTGAAGGTCTCCTGCAGGGCTT
CTGGAGGCACCTTC------------
AGCAAGTATGCTATCAGCTGGGTGCGACAGGCCCCTGGACAAGGGCTTGA
GTGGATGGGAGGGATCATCCCTATT------TTTGGTACACCCAATTACGCACAGAAGTTCCAG---

GGCAGAGTC
ACGATTACCGCGGACGAATCCACGACCACAGCCTACATGGAACTGAGTAGCCTCACATCTGAGGACACTGCCGTG
T
ATTATTGTGCGAGAGGAACTGGGGACCACACCACGGTGGTGATTCCTTTCGACTACTGGGGCCCGGGAACCCTGG
T
C
>38-/D-/27+|F3-VH1/3|Wk08
CAGGTGCAGCTGGTGCAGTCTGGGGCT---
GAGGTGAAGAAGCCTGGGTCCTCGGTGAAGGTCTCCTGTAGGGCTT
CTGGAGGCACCTTC------------
AGCAAGTATGCTATTAGCTGGATGCGACAGGCCCCTGGACAAAGACTTGA
GTGGATGGGAGGGATTACCCCCATC------TTTGGCACGCCAAATTACGCACACAAATTTCAG---
GGCAGACTC
ACCATTACCGCGGACGACTCCACGAGCACAGCGTACATGGAACTGAGCGGCCTGAGATCTGAGGACACGGCCGTC
T
ATTACTGTGCGAGAGGAACTGGGGACCACACTACGGTGGTGACTCCTTTCGACTACTGGGGCCAGGGAACNNNNNN
N
N
>38-/D-/27+|F4-VH1/3|Wk08
NNNGTGCAGCTGGTACAGTCTGGGGCT---
GAACTGAAGAAGCCTGGGTCCTCGGTGAAGGTCTCCTGCAGGGCTT
CTGGCGGCACCTTC------------
AGCAAATATGCTATCAGCTGGGTGCGACAGGCCCCTGGACAAGGGCTTGA
GTGGATGGGAGGGATCATCCCTATC------TTTGGTACACCCAATTACGCACAGAAGTTCCAG---
GGCAGAGTC
ACCATTACCGCGGACGAATCCACGAGCACAGCCTACATGGAACTGAGCAGCCTCAGATCTGAAGACACTGCCGTT
T
ATTACTGTGCGAGAGGAACTGGGGACCACACTACGGTGGTAACTCCTTTCGACTACTGGGGCCAGGGAACCCTGG
T
C
>38-/D-/27+|F11-VH1/3|Wk08
GAGGTGCAGCTGGTGCAGTCTGGGGCT---
GAGGTGAAGAAGCCTGGGTCCTCGGTGAAGGTCTCCTGCAGGGCTT
CTGGAGGCACCTTC------------
AGCAAGTCTGCTATCAGCTGGGTGCGACAGGCCCCTGGACAAGGGCTTGA
GTGGATGGGAGGGATCATCCCTATC------TTTGGTACAGGGAATTACGCACAAAAATTCCAG---
GGCAGGGTC
ACGATTACCGCGGACGAATCCACGACCACAGCCCACATGGAGCTGAGTAGCCTGAGATCTGAGGACACGGCCGTA
T
ATTACTGTGCGAGAGGAAGTGGGGACCACACTACGGTGCTAACTCCTTTCGACTACTGGGGCCAGGGAACCCTGG
T
C
>38-/D-/27+|G3-VH1/3|Wk08
GAGGTGCAGCTGGTGGAGTCTGGGGCT---
GAACTGAAGAAGCCTGGGTCCTCGGTGAAGGTCTCCTGCAGGGCTT
CTGGAGGCACGTTC------------

AGCAAATATGCTATCAGCTGGGTGCGACAGGCCCCTGGACAAGGGCTTGA
GTGGATGGGAGGGATCATCCCTATC------TTTGGTACACCCAATTACGCACAGAAGTTCCAG---
GGCAGAGTC
ACCATTAGCGCGGACGAATCCACAAACACAGCCTACTTGGAACTGAGCAGCCTCAGATCTGAAGACACTGCCGTA
T
ATTACTGTGCGAGAGGAACTGGGGACCACACTACGGTGGTAACTCCTTTCGACTACTGGGGCCAGGGAACCCTAG
T
C
>38-/D-/27+|G10-VH1/3/5|Wk08
NNNNNNNAGCTGGTGCAGTCTGGGGCT---
GAGGTGAAGAAGCCTGGGTCCTCGGTGAAGGTCTCCTGCAGGGCTT
CTGGAGGCACCTTC------------
AGCAAGTATGCCATCAGCTGGGTGCGACAGGCCCCTGGACAAGGGCTTGA
GTGGATGGGAGGGATCATCCCTATC------TTTGGTACACCCAATTACGCACAGAAGTTCCAG---
GGCAGAGTC
ACGATTACCGCGGACGAATCTACGAGCACAGCCTACATGGAACTGAGCAGCCTAAGATCTGAGGACACTGCCATA
T
ATTACTGTGCGAGAGGAACTGGGGACCACACTACGGTGGTAACTCCTTTCGACTACTGGGGCCAGGGAACCCTGG
T
C
>38-/D-/27+|G11-VH1/3/5|Wk08
CAGGTGCAGCTGGTGCAGTCTGGGGCA---
GAGGTGAAGAAGCCTGGGTCCTCGGTGAAGGTCTCCTGCAGGGCTT
CTGGAGGCACCTTC------------
AGCAAGTATGCTATCAGCTGGGTGCGACAGGCCCCTGGACAAGGGCTTGA
GTGGATGGGAGGGATCATCCCTATT------TTTGGTACACCCAATTACGCACAGAGGTTCCAG---
GGCAGAGTC
ACGATTACCGCGGACGAATCCACGAGCACAGCCTACATGGAACTGAGCAGCCTCAGATCTGAGGACACTGCCGTA
T
ATTACTGTGCGAGAGGAACTGGGGACCACACTACGGTGGTCACTCCTTTCGACTACTGGGGCCAGGGAACCCTGG
T
C
>38-/D-/27+|H2-VH1/3|Wk08
NNGGTGCAGCTGGTACAGTCTGGGGCT---
GAACTGAAGAAGCCTGGGTCCTCGGTGAAGATCTCCTGCAGGGCTT
CTGGAGGCACCTTC------------
AGCAAGTATGCTATCAGCTGGGTGCGACAGGCCCCTGGACAAGGGCTTGA
GTGGATGGGAGGGATCATCCCTATC------TTTGGTACACCCAATTATGCACAGAAGTTCCAG---
GGCAGAGTC
ACCATTACCGCGGACGAATCCACGACCACAGCCTACATGGAGATGAGCAGCCTCAGATCTGAAGACACTGCCTTG
T
ATTACTGTGCGAGAGGAACTGGGGACCACACTACGGTGGTAACTCCTTTCGACTACTGGGGCCAGGGAACCCTGG
T
C
>38-/D-/27+|H4-VH1/3|Wk08
GAGGTGCAGCTGGTACAGTCTGGGGCT---

GAACTGAAGAAGCCTGGGTCCTCGGTGAAGATCTCCTGCAGGGCTT
CTGGAGGCACCTTC------------
AGCAAGTATGCTATCAGCTGGGTGCGACAGGCCCCTGGACAAGGGCTTGA
GTGGATGGGAGGGATCATCCCTATC------TTTGGTACACCCAATTATGCACAGAAGTTCCAG---
GGCAGAGTC
ACCATTACCGCGGACGAATCCACGAGCACAGCCTACATGGAACTGAGCAGCCTCAGATCTGAAGACACAGCCATA
T
ACTACTGTGCGAGAGGAACTGGGGACCACACTACGGTGGTAACCCCTTTCGACTACTGGGGCCAGGGAACCCTGG
T
C
>38-/D-/27+|H6-VH1/3/5|Wk08
NNNNNNNAGCTGGTGCAGTCTGGAGCA---
GAGGTGAAGAAGCCTGGGTCCTCGGTGAAGGTCTCCTGCAGGGCTT
CTGGAGGCACCTTC------------
AGCAAGTATGCTATCAGCTGGGTGCGACAGGCCCCTGGACAAGGGCTTGA
GTGGATGGGAGGGATCATCCCTATC------TTTGGTACACCCAATTACGCACAAAAGTTCCAG---
GGCAGAGTC
ATGATTACCGCGGACGAATCCACGAGCACAGCCTACATGGAACTGAGCAGCCTGAGATCTGAGGACACTGCCATA
T
ATTACTGTGCGAGAGGAACTGGGGACCACACTACGGTGGTAACTCCTTTCGACTATTGGGGCCAGGGAACCCTGG
T
C
>38-/D-/27+|H5-VH1/5|Wk08
NNGGTGCAGCTGGTACAGTCTGGGGCT---
GAGGTGAAGAAGCCTGGGTCCTCGGTGAAGGTCTCCTGCAGAGCTT
CTGGAGGGACCTTC------------
AGCAAGTATGCTATCAGTTGGGTGCGACAGGCCCCTGGACAGGGGCTTGA
GTGGATGGGAGGGATCATCCCTATC------TTCGGTACACCCAATTACGCACAGAAGTTCCAG---
GGCAGAGTC
ACGATTACCGCGGACGAATCCACGAGCACAGCCTACATGGAACTGAGTAGCCTCACATCTGAGGACACAGCCGTT
T
ATTACTGTGCGAGAGGAACTGGGGACCACACTACGGTGGTAACTCCTTTCGATTACTGGGGCCANNNNNNNNNNN
N
N
>38-/D-/27+|G2-VH1|Wk08
NNGTGCAAGCTGGTACAGTCTGGGGCT---
GAACTGAAGAAGCCTGGGTCCTCGGTGAAGATCTCCTGCAGGGCTT
CTGGAGGCACCTTC------------
AGCAAGTATGCTATCAGCTGGGTGCGACAGGCCCCTGGACAAGGGCTTGA
GTGGATGGGAGGGATCATCCCTATC------TTTGGTACACCCAATTATGCACAGAAGTTCCAG---
GGCAGAGTC
ACCATTACCGCGGACGAATCCACGACCACAGCCTACATGGAGATGAGCAGCCTCAGATCTGAAGACACTGCCTTG
T
ATTACTGTGCGAGAGGAACTGGGGACCACACTACGGTGGTAACTCCTTTCGACTACTGGGGCCAGGGAACCCTGG
T
C

>38-/D-/27+|A10-VH1/3/5|Wk14
GAGGTGCAGCTGGTGCAGTCTGGGGCT---
GAGGTGAAGAAGCCTGGGTCCTCGGTGAAGGTCTCCTGCAGGGCTT
CTGGAGGCACCTTC------------
AGCAAGTATGCTATCAGCTGGGTGCGACAGGCCCCTGGACAGGGGCTTGA
GTGGATGGGAGGGATCATCCCTATC------TTTGGTACACCCAATTACGCACAGAAGTTCCAG---
GGCAGAGTC
ACGATTAGCGCGGACGAATCTACGAGCACAGCCTACATGGAACTGAACAGCCTGAGATCTGAGGACACGGCCATA
T
ACTACTGTGCGAGAGGAACAGGGGACCACACTACGGTGGTAACTCCTTTCGACTACTGGGGCCAGGGAACCCTGG
T
C
>38-/D-/27+|B5-VH1/5|Wk14
NNGGTGCAGCTGGTGCAGTCTGGGGCT---
GAGGTGAAGAAGCCTGGGTCCTCGGTGAAGGTCTCCTGCAGAGCTT
CTGGAGGGACCTTC------------
AGCAAGTATGCTATCAGCTGGGTGCGACAGGCCCCTGGACAAGGGCTTGA
GTGGATGGGAGGGATCATCCCTATC------TTTGGTACACCCAATTACGCACAGAAGTTCCAG---
GGCAGAGTC
ACGATTACCGCGGACGAATCCACGAGCACAGCCTACATGGAACTGAGCAGCCTCACATCTGAGGACACAGCCGTT
T
ATTACTGTGCGAGAGGAACTGGGGACCACACTACGGTGGTAACTCCTTTCGACTACTGGGGCCAGGGAACCCTGG
T
C
>38-/D-/27+|B7-VH1/3|Wk14
NNGGTGCAGCTGGTGCAGTCTGGGGCT---
GAACTGAAGAAGCCTGGGTCCTCGGTGAAGGTCTCCTGCAGGGCTT
CTGGAGGCACATTC------------
AGCAAATATGCTATCAGCTGGCTGAGACAGGCCCCTGGACAAGGGCTTGA
GTGGATGGGAGGGATCATCCCTATC------TTTGGTACACCCAATTACGCACAGAAGTTCCAG---
GGCAGGGTC
ACCATTACCGCGGACGAGTCCACAAACACAGCCTACTTGGAACTGAGCAGCCTCAGATCTGAAGACACTGCCGTT
T
ATTACTGTGCGCGAGGAACTGGGGACCACACTACGGTGGTAACTCCTTTCGACTACTGGGGCCAGGGAACCCTGG
T
C
>38-/D-/27+|B11-VH1/3/5|Wk14
NNGGTGCAGCTGGTGCAGTCTGGGGCT---
GAGGTGAAGAAGCCTGGGTCCTCGGTGAAGGTCTCCTGCAGGGCTT
CTGGAGGCACCTTC------------
AGCAAGTATGCTCTCAGCTGGGTGCGACAGGCCCCTGGACAAGGTGTTGA
GTGGATGGGAGGGATCATCCCAATC------TTTGGAACAGCAAACTACGCACAAAAGTTCCAG---
GGCAGAGTC
ACGATTACCGCGGACGAATCCACGAGCACAGCCTACATGGAGCTGAGCAGCCTGAGATCTGAGGACACGGCCGTG
T
ATTACTGTGCGAGAGGAACTGGGGACCACACTACGGTGGTAACTCCTTTCGACTACTGGGGCCAGGGAACCCTGG

T
C
>38-/D-/27+|C2-VH1/3/5|Wk14
GAGGTGCAGCTGGTGCAGTCTGGGGCT---
GAGGTGAAGAAGCCTGGGTCCTCGGTGAAGGTCTCCTGCAGGGCTT
CTGGAGGCACCTTC------------
AGCAAATATGCTATCAGTTGGGTGCGACAGGCCCCTGGACAAGGGCTTGA
GTGGATGGGAGGGATCATCCCTATC------TTTGGTACGGCAAACTACGCACAGAAGTTCCAG---
GGCAGAGTC
ACGATTACCGCGGACGAATCCACGAGCACAGCCTACATGGAGGTGAGCAGCCTGAGATCTGAGGACACGGCCGTG
T
ATTACTGTGCGAGAGGAACTGGGGACCACACTACGGTGGTAACTCCTTTCGACTACTGGGGCCAGGGAACCCTGG
T
C
>38-/D-/27+|C4-VH1/3/5|Wk14
GAGGTGCAGCTGGTGCAGTCTGGGGCT---
GAGGTGAAGAAGCCTGGGTCCTCGGTGAAGGTCTCTTGCAGGGCTT
CTGGAGGCACCTTC------------
AGTAAGTATGCTATCAGCTGGGTGCGACAGGCCCCTGGACAAGGCCTTGA
GTGGATGGGAGGAATTATCCCCATC------TTTGGTACACCAAATTACGCACAAAAGTTCCAG---
GGCAGAGTC
ACAATTACCGCGGACGAATCCACGAGCACAGCCTACATGGAACTGAGCAGCCTGACATCTGAGGACACTGCCGTA
T
ACTACTGTGCGAGAGGAACTGGGGACCACACAACGGTGGTAACTCCTTTCGACTACTGGGGCCAGGGAACCCTGG
T
C
>38-/D-/27+|A6-VH1/3|Wk14
GAGGTCCAGCTGGTGGAGTCTGGGGTT---
GAACTGAAGAAGCCTGGGTCCTCGGTGAAGGTCTCCTGCAGGGCTT
CTGGAGGCACCTTC------------
AGCAAGTATGCTATCAGCTGGGTGCGACAGGCCCCTGGACAAGGGCTTGA
GTGGATGGGAGGGATCATCCCTATC------TTTGGTACACCCAATTACGCACAGAAGTTCCAG---
GGCAGAGTC
ACCATTACCGCGGACGAATCCACGAGCACAGCCTACATGGAACTGAGCAGCCTCAGATCTGAAGACACTGCCATA
T
ATTACTGTGCGAGAGGAACTGGGGACCACACTACGGTGGTAACTCCTTTCGACTACTGGGGCCAGGGAACCCTGG
T
C
>38++/D-/27+|A4-VH1/3/5|Wk26
GAGGTGCAGCTGGTGGAGTCTGGGGCT---
GAGGTGAAGAAGCCCGGGTCCTCGGTGAAGGTCTCCTGCAGGGCTT
CTGGAGGCACCTTC------------
AGCAAATATGCTGTCAGTTGGCTGCGACAGGCCCCTGGACAGGGGCTTGA
GTGGATGGGGGGAATCATCCCTATT------TTTGGTACAGCAAACTACGCACAAAAGTTCCAG---
GGCAGAGTC
ACGATTACCGCGGACGAATCCACGAATACAGCCTACATGGAGGTGAGTAGTCTGACATCTGAAGACACGGCCGTT

T
ACTATTGCGCGAGAGGAACTGGGGACCACACTACGGTGGTGACTCCTTTCGACTACTGGGGCCAGGGAACCCTGG
T
C
>38++/D-/27+|B2-VH1/3|Wk26
NNGGTGCAGCTGGTGCAGTCTGGGGCT---
GAACTGAAGAAGCCTGGATCCTCGGTGAAGGTCTCCTGCAGGGCTT
CTGGCGGCACCTTC------------
AGCAAATATGCTATCAGCTGGGTGCGACAGGCCCCTGGACAAGGGCTTGA
GTGGATGGGAGGGATCATCCCCATC------TTCGGTACACCCAATTACGCACAGAAGTTCCAG---
GGCAGAGTC
ACCATTACCGCGGACGAATCCACGAGCACAGCCTACATGGAACTGAGCAGCCTCAGATCTGAAGACACTGCCGTT
T
ATTACTGTGCGAGAGGAACTGGGGACCACACTACGGTGGTAACTCCTTTCGACTACTGGGGCCAGGGAACCCTGG
T
C
>38++/D-/27+|B7-VH1/3/5|Wk26
NNGGTGCAGCTGGTACAGTCTGGGGCT---
GAGGTGAAGAAGCCTGGGTCCTCGGTGAAGGTCTCCTGCAGGGCTT
CTGGAGGCACCTTC------------
AGCAAGTATGCTATCAGCTGGGTGCGACAGGCCCCTGGACAGGGGCTTGA
ATGGATGGGAGGGATCATCCCTATC------TTTGGTACACCCAATTACGCACAGAAGTTCCAG---
GGCAGAGTC
ACGATTACCGCGGACGAATCTACGAACACAGCCTACATGGAACTGAGCAGCCTGAGATCTGAAGACACTGCCGTA
T
ATTACTGTGCGAGAGGAACTGGGGACCATACTACGGTGGTAACTCCTTTCGACTACTGGGGCCAGGGAACCCTGG
T
C
>38++/D-/27+|B11-VH1/3|Wk26
GAGGTGCAGCTGGTGGAGTCTGGGGCT---
GAACTGAAGAAGCCTGGGTCCTCGGTGAAGGTCTCCTGCAGGGCTT
CTGGAGGCACCTTC------------
AGCAAGTATGCTATCAGCTGGGTGCGACAGGCCCCTGGACAAGGGCTTGA
GTGGATGGGAGGGATCATCCCTATC------TTTGGTACACCCAATTACGCACAGAAGTTCCAG---
GGCAGAGTC
ACCATTACTGCGGACGAATCCACGAGCACAGCCTACATGGAACTGAGCAGCCTCAGATCTGAAGACACTGCCGTA
T
ATTACTGTGCGAGAGGAACTGGGGACCACACTACGGTGGTAACTCCTTTCGACTACTGGGGCCAGGGAACCCTGG
T
C
>38++/D-/27+|D3-VH1/3/5|Wk26
GAGGTTCAGCTGGTGGAGTCTGGGGCT---
GAGGTGAAGAAGCCTGGGTCCTCGGTGAAGGTCTCCTGCAGGGCTT
CTGGAGGCACTTTC------------
AGCAAGTATGCTATCAGCTGGGTGCGACAGGCCCCTGGACAAGGGCTTGA
GTGGATGGGAGGGATCATCCCTATC------TTTGGTACACCAAACTACGCACAAAAGTTCCAG---

GGCAGAGTC
ACGATTACCGCGGACGAATCCACGAGCACAGCCTATATGGAATTGAGCAGCCTGAGATCTGAGGACACGGCCGTA
T
ATTACTGTGCGAGAGGAACTGGGGACCACACTACGGTGGTAACTCCTTTCGACTACTGGGGCCAGGGAACCCTGG
T
C
>38++/D-/27+|D10-VH1/3/5|Wk26
GAGGTGCAGCTGGTGCAGTCTGGGGCT---
GAGGTGAAGAAGCCTGGGTCCTCGGTGAAGGTCTCCTGCAGGGCTT
CTGGAGGCACCTTC------------
AGCAAGTATGCTATCAGCTGGGTGCGACAGGCCCCTGGACAGGGGCTTGA
ATGGATGGGAGGGATCATCCCTATC------TTTGGTACACCCAATTACGCACAGAAGTTCCAG---
GGCAGAGTC
ACGATTACCGCGGACGAATCTACGAACACAGCCTACATGGAACTGAGCAGCCTGAGATCTGAAGACACTGCCGTA
T
ATTACTGTGCGAGAGGAACTGGGGACCATACTACGGTGGTAACTCCTTTCGACTACTGGGGCCAGGGAACCCTGG
T
C
>38++/D-/27+|D11-VH1/3|Wk26
NNGGTGCAGCTGGTACAGTCTGGGGCT---
GAACTGAAGAAGCCTGGGTCCTCGGTGAAGGTCTCCTGCAGGGCTT
CTGGAGGCACCTTC------------
AGCAAGTATGCTATCAGCTGGGTGCGACAGGCCCCTGGACAAGGGCTTGA
ATGGATGGGAGGGATCATCCCTATC------TTTGGTACACCCAATTACGCACAGAAGTTCCAG---
GGCAGAGTC
ACCATTACCGCGGACGAATCCACGAGCACAGCCTACATGGAACTGAGCAGCCTCAAATCTGAAGACACTGCCGTC
T
ATTACTGTGCGAGAGGAACTGGGGACCACACTACGGTGGTAACTCCTTTCGACTACTGGGGCCAGGGAACCCTGG
T
C
>38-/D-/27+|G11-VH1/3|Wk26
GAGGTGCAGCTGGTGCAGTCTGGGGCT---
GAGGTGAAGAAGCCTGGGTCCTCGGTGAAGGTCTCCTGCAGGGCTT
CTGGAGGCACCTTC------------
AGCAAGTATGCCATCAGCTGGGTGCGACAGGCCCCTGGACAAGGGCTTGA
GTGGATGGGAGGGATCATCCCTATC------TTTGGTACACCCAATTACGCACAGAAGTTCCAG---
GGCAGAGTC
ACGATTACCGCGGACGAATCCACGAGCACAGCCTACATGGAACTGAGCAGCCTAAGATCTGAGGACACTGCCATA
T
ATTACTGTGCGAGAGGAACTGGGGACCACACTACGGTAGTAACTCCTTTCGACTACTGGGGCNNNNNNNNNNNNN
N
N
>38++/D-/27+|A2-VH1/3/5|Wk26
GAGGTGCAGCTGGTGCAGTCTGGGGCT---
GAGGTGAAGAAGCCTGGGTCCTCGGTGAAGGTCTCCTGCAGGGCTT
CTGGAGGCACCTTC------------

AGCAAGTATGCTATCAGCTGGGTGCGACAGGCCCCTGGACAGGGGCTTGA
ATGGATGGGAGGGATCATCCCTATC------TTTGGTACACCCAATTACGCACAGAAGTTCCAG---
GGCAGAGTC
ACGATTACCGCGGACGAATCTACGAACACAGCCTACATGGAACTGAGCAGCCTGAGATCTGAAGACACTGCCGTA
T
ATTACTGTGCGAGAGGAACTGGGGACCATACTACGGTGGTAACTCCTTTCGACTACTGGGGCCAGGGAACCCTGG
T
C
>38++/D-/27+|C8-VH1/3/5|Wk26
CAGGTGCAGCTGGTGGAGTCTGGGGCT---
GAGGTGAAGAAGCCTGGGTCCTCGGTGAGGGTCTCCTGCAGGGCTT
CTGGAGGCACCTTC------------
AGCAACTATTTTATCGGCTGGCTGCGACAGGCCCCTGGACAAGGGCTTGA
GTGGATGGGAGGGATCATCCCTATC------TTTCGTGCTGCAAACTTCGCACAAAAGTTCCAG---
GGCAGAGTC
AACATTACCGCGGACGACTCCACGAGCACAGCCTACATGGAACTGAGCGGCCTGAGATCTGATGACACGGCCGTG
T
ATTACTGTGCGAGAGGAACTGGGGACCACACCACGGTGGTAACTCCTTTCGACTTCTGGGGCNNNNNNNNNNNNN
N
N
>38++/IGD-/27+|A2-VH1/3/5|Wk36
GAGGTGCAGCTGGTGCAGTCTGGGGCT---
GAGGTGAAGAAGCCTGGGTCCTCGGTGAAGGTCTCCTGCAGGGCTT
CTGGAGGCACCTTC------------
AGCAAGTATGCTATTAGTTGGGTGCGACAGGCCCCTGGACAAGGGCTTGA
GTGGATGGGAGGGATCATCCCTATC------TTTGGTACACCCAATTACGCACAGAAGTTCCAG---
GGCAGAGTC
ACGATTACCGCGGACGAATCCACGAGCACATCCTACATGGAGCTGAGCAGCCTCAGATCTGAAGACACTGCCATA
T
ATTACTGTGCGAGAGGAACTGGGGACCACACTACGGTGGTAACTCCTTTCGACTATTGGGGCCAAGGCACCCTGG
T
C
>38++/IGD-/27+|A4-VH1/3/5|Wk36
GAGGTGCAGCTGGTGCAGTCTGGGGCT---
GAGGTGAAGAGGCCTGGGTCCTCGGTGAAGGTCTCCTGCAGGGCTT
CTGGAGGCACCTTC------------
AGCAAGTTTGCTATCAGCTGGGTGCGACAGGCCCCTGGACAAGGGCTTGA
GTGGATGGGAGGGATCATCCCTATT------TTTGGGACAGCAAACTACGCACAAAAGATGCAG---
GGCAGAGTC
ACGATAACCGCGGACGAATCCACGAACACAGCCTACATGGAGCTGGGCAGCCTGACATCTGAGGACACGGCCGTA
T
ATTACTGTGCGAGAGGAACTGGGGACCACACAACGGTGGTGACTCCTTTCGACTACTGGGGCCAAGGCACCCTGG
T
C
>38++/IGD-/27+|A6-VH1/3/5|Wk36
GAGGTGCAGCTGGTGCAGTCTGGGGCT---

GAGGTAAAGAAGCCTGGGTCCTCGGTGAAGGTCTCCTGCAAGGCTT
CTGGAGGCACCTTC------------
AGCAAGTATGCTATCAACTGGGTGCGACAAAGTCCTGGACAAGGGCTTGA
GTGGATGGGAGGGATCATCCCTATC------TTTGGTACACCAAACTACGCACAAAAGTTCCAG---
GGCAGAGTC
ACGATTACCGCGGACGAATCTACGAGCACAGCCTACATGGAGGTGAGCAGCCTGAGATCTGAAGACACGGCCGTT
T
ATTATTGTGCGAGAGGAACTGGGGACCACACAACGGTGGTAACTCCTTTCGACTACTGGGGCCAAGGCACCCTGG
T
C
>38++/IGD-/27+|B2-VH1/3/5|Wk36
GAGGTGCAGCTGGTGCAGTCTGGGGCT---
GAGGTGAAGAAGCCTGGGTCCTCGGTGAAAATCTCCTGCAGGGCTT
CTGGAGGCACCTTC------------
AGCAAGTATGCTATCAGTTGGGTGCGACAGGCCCCTGGACAAGGGCTTGA
GTGGATGGGAGGGATCATCCCTATC------TTTGGTACACCCAATTACGCACAGAAGTTCCAG---
GGCAGAGTC
ACGATTACCGCGGACGAATCCACGAGTACAGCCTACATGGAACTGAGCAGCCTGAGATCTGAGGACACTGCCGTA
T
ATTACTGTGCGAGAGGAACTGGGGACCACACTACGGTGGTAACTCCTTTCGACTACTGGGGCCAAGGCACCCTGG
T
C
>38++/IGD-/27+|B8-VH1/3/5|Wk36
GAGGTGCAGCTGGTGCAGTCTGGGGCT---
GAGGTGAAGAAGCCTGGGTCCTCGGTGAAGGTCTCCTGCAGGGCTT
CTGGAGGCACCTTC------------
AGCAAGTATGCTATCAGCTGGGTGCGACAGGCCCCTGGACAGGGGCTTGA
GTGGATGGGAGGGATCATCCCTATC------TTTGGTACACCCAATTACGCACAGAAGTTCCAA---
GGCAGAGTC
ACGATTACCGCGGACGAATCCACGAGCACAGCCTACATGGAACTGAGCAGCCTGAGATCTGAGGACACTGCCGTG
T
ATTTCTGTGCGAGAGGAACTGGGGACCACACTACGGTGGTAACTCCTTTCGACTACTGGGGCCAAGGCACCCTGG
T
C
>38++/IGD-/27+|A10-VH1/3/5|Wk36
NNGGTGCAGCTGGTGCAGTCTGGGGCT---
GAGGTGAAGAAGCCTGGGTCCTCGGTGAAGGTCTCCTGCAGGGCTT
CTGGAGGCACCTTC------------
AGCAAGTATGCTATCAGCTGGGTGCGACAGGCCCCCGGACAGGGGCTTGA
GTGGATGGGAGGGATCATCCCTATC------TTTGGTACACCCAATTACGCACAGAAGTTCCAG---
GGCAGAGTC
ACAATTACCGCGGACGAATCCACGAGCACAGCCTACATGGAACTGAGCAGCCTGAGATCTGAAGACACTGCCGTA
T
ATTACTGTGCGAGAGGAACTGGGGACCATACTACGGTGGTTACTCCTTTCGACTACTGGGGCCAGGGCACCCTGG
T
C

>38++/D-/27+|A10-VH1/3|Wk52
NAGGTTCAGCTGGTGGAGTCTGGGGCT---
GAACTGAAGAAGCCTGGGTCCTCGGTGAAGGTCTCCTGCAGGGCTT
CTGGAGGCACATTC------------
AGCAAATATGCTATCAGCTGGCTGAGACAGGCCCCTGGACAAGGGCTTGA
GTGGATGGGAGGGATCATCCCTATC------TTTGGTACACCCAATTACGCACAGAAGTTCCAG---
GGCAGGGTC
ACCATTACCGCGGACGAGTCCACAAACACAGCCTACTTGGAACTGAGCAGCCTCAGATCTGAAGACACTGCCGTT
T
ATTACTGTGCGCGAGGAACTGGGGACCACACTACGGTGGTAACTCCTTTCGACTACTGGGGCCAGGGAACCCTGG
T
C
>38++/D-/27+|A11-VH3|Wk52
GAGGTGCAGCTGGTGGAGTCTGGGGCT---
GAGGTGAAGAAGCCTGGGTCCTCGGTGAAGGTCTCCTGCAGGGCTT
CTGGAGGCACTTTC------------
AGCAAGTATGCTATCAGCTGGGTGCGACAGGCCCCTGGACAAGGGCTTGA
GTGGATGGGAGGGATCATCCCTATC------TTTGGTACAGCAAACTACGCACAAAAGTTCCAG---
GGCAGAGTC
ACGATTACCGCGGACGAATCCACGAGCACAGCCTACATGGAGCTGAGCAGCCTGAGATCTGAGGACACGGCCGTG
T
ATTACTGTGCGAGAGGAACTGGGGACCACACTACGGTGGTAACTCCTTTCGACTACTGGGGCCAGGGAACCCTGG
T
C
>38++/D-/27+|B1-VH1|Wk52
NNGGTGCAGCTGGTGCAGTCTGGGGCT---
GAGGTGAAGAAGCCTGGGTCCTCGGTGAAGGTCTCCTGCAGGGCTT
CTGGAGGCACCTTC------------
AGCAAGTATGCTATCAGCTGGGTGCGACAGGCCCCTGGACAGGGGCTTGA
GTGGATGGGAGGGATCATCCCTATC------TTTGGTACACCCAATTACGCACAGAAGTTCCAG---
GGCAGAGTC
ACGATTACCGCGGACGAATCCACGAGCACAGCCTACATGGAGCTGAGCAGCCTGAGATCTGAGGACACTGCCGTA
T
ATTACTGTGCGAGAGGAACTGGGGACCACACTACGGTGGTAACTCCTTTCGACTACTGGGGCCAGGGAACCCTGG
T
C
>38++/D-/27+|B1-VH3|Wk52
GAGGTTCAGCTGGTGGAGTCTGGGGCT---
GAGGTGAAGAAGCCTGGGTCCTCGGTGAAGGTCTCCTGCAGGGCTT
CTGGAGGCACCTTC------------
AGCAAGTATGCTATCAGCTGGGTGCGACAGGCCCCTGGACAGGGGCTTGA
GTGGATGGGAGGGATCATCCCTATC------TTTGGTACACCCAATTACGCACAGAAGTTCCAG---
GGCAGAGTC
ACGATTACCGCGGACGAATCCACGAGCACAGCCTACATGGAGCTGAGCAGCCTGAGATCTGAGGACACTGCCGTA
T
ATTACTGTGCGAGAGGAACTGGGGACCACACTACGGTGGTAACTCCTTTCGACTACTGGGGCCAGGGAACCCTGG

T
C
>38++/D-/27+|B4-VH1|Wk52
NNGGTGCAGCTGGTACAGTCTGGGGCT---
GAGGTGAAGAAGCCTGGGTCCTCGGTGAAGGTCTCCTGCAGGGCTT
CTGGAGGCACCTTC------------
AGCAAGTATGCTATCAGCTGGGTGCGACAGGCCCCTGGACAGGGGCTTGA
GTGGATGGGAGGGATCATCCCTATC------TTTGGTACACCCAATTACGCACAGAAGTTCCAG---
GGCAGAGTC
ACAATTACCGCGGACGAATCCACGAGCACAGCCTACATGGAACTGAGCAGCCTGAGATCTGAAGACACTGCCGTA
T
ATTACTGTGCGAGAGGAACTGGGGACCATACTACGGTGGTAACTCCTTTCGACTACTGGGGCCAGGGAACCCTGG
T
C
>38++/D-/27+|B4-VH3|Wk52
NNNNNNNNGCTGGTGGAGTCTGGGGCT---
GAGGTGAAGAAGCCTGGGTCCTCGGTGAAGGTCTCCTGCAGGGCTT
CTGGAGGCACCTTC------------
AGCAAGTATGCTATCAGCTGGGTGCGACAGGCCCCTGGACAGGGGCTTGA
GTGGATGGGAGGGATCATCCCTATC------TTTGGTACACCCAATTACGCACAGAAGTTCCAG---
GGCAGAGTC
ACAATTACCGCGGACGAATCCACGAGCACAGCCTACATGGAACTGAGCAGCCTGAGATCTGAAGACACTGCCGTA
T
ATTACTGTGCGAGAGGAACTGGGGACCATACTACGGTGGTAACTCCTTTCGACTACTGGGGCCAGGGAACCCTGG
T
C
>38++/D-/27+|C10-VH1|Wk52
NNGGTGCAGCTGGTACAGTCTGGGGCT---
GAGGTGAAGAAGCCTGGGTCCTCGGTGAAGGTCTCCTGCAGGGCTT
CTGGAGGCACCTTC------------
AGCAAGTATGCTATCAGCTGGGTGCGGCAGGCCCCTGGACAAGGGCTTGA
GTGGATGGGAGGGATCATCCCTATC------TTTGGTACACCCAATTACGCACAGAAGTTCCAG---
GGCAGAGTC
ACGATTACCGCGGACGAATCCACGAGCACAGCCTACATGGAACTGAGCAGCCTGAGATCTGAGGACACTGCCGTG
T
ATTACTGTGCGAGAGGAACTGGGGACCACACTACGGTGGTAACTCCTTTCGACTACTGGGGCCAGGGAACCCTGG
T
C
>38++/D-/27+|C10-VH3|Wk52
GAGGTGCAGCTGGTGGAGTCTGGGGCT---
GAGGTGAAGAAGCCTGGGTCCTCGGTGAAGGTCTCCTGCAGGGCTT
CTGGAGGCACCTTC------------
AGCAAGTATGCTATCAGCTGGGTGCGGCAGGCCCCTGGACAAGGGCTTGA
GTGGATGGGAGGGATCATCCCTATC------TTTGGTACACCCAATTACGCACAGAAGTTCCAG---
GGCAGAGTC
ACGATTACCGCGGACGAATCCACGAGCACAGCCTACATGGAACTGAGCAGCCTGAGATCTGAGGACACTGCCGTG

T
ATTACTGTGCGAGAGGAACTGGGGACCACACTACGGTGGTAACTCCTTTCGACTACTGGGGCCAGGGAACCCTGG
T
C
>38-/D-/27+|G6-VH1/3|Wk52
GAGGTGCAGCTGGTGGAGTCTGGGGCT---
GAACTGAAGAAGCCTGGGTCCTCGGTGAAGGTCTCCTGCAGGGCTT
CTGGAGGCACCTTC------------
AGCAAGTATGCTATCAGCTGGGTGCGACAGGCCCCTGGACAAGGGCTTGA
GTGGATGGGAGGGATCATTCCTATC------TTTGGTACACCCAATTACGCACAGAAGTTCCAG---
GGCAGAGTC
ACCATTACCGCGGACGAATCCACGACCACAGCCTACTTGGAACTGAGCAGCCTCAGATCTGAAGACACTGCCGTG
T
ATTATTGTGCGAGAGGAACTGGGGACCACACTACGGTGGTAACTCCTTTCGACTACTGGGGCCAGGGAACCCTGG
T
C
>38-/D-/27+|H6-VH1/3|Wk52
GAGGTGCAGCTGGTGGAGTCTGGGGCT---
GAACTGAAGAAGCCTGGGTCCTCGGTGAAGATCTCCTGCAGGGCTT
CTGGAGGCACCTTC------------
AGCAAGTATGCTATCAGTTGGGTGCGACAGGCCCCTGGACAAGGGCTTGA
GTGGATGGGAGGGATCATCCCTATC------TTTGGTACACCCAATTATGCACAGAAGTTCCAG---
GGCAGAGTC
ACCATTACCGCGGACGAATCCACGAGCACAGCCTACATGGAACTGAGCAGCCTCAGATCTGAAGACACTGCCGTA
T
ATTACTGTGCGAGAGGAACTGGGGACCACACTACGGTGGTAACTCCTTTCGACTACTGGGGCCAGGGAACCCTGG
T
C
>38++/D-/27+|A11-VH5|Wk52
NNNNNNTAGCTGGTGCAGTCTGGAGCA---
GAGGTGAAGAAGCCTGGGTCCTCGGTGAAGGTCTCCTGCAGGGCTT
CTGGAGGCACTTTC------------
AGCAAGTATGCTATCAGCTGGGTGCGACAGGCCCCTGGACAAGGGCTTGA
GTGGATGGGAGGGATCATCCCTATC------TTTGGTACAGCAAACTACGCACAAAAGTTCCAG---
GGCAGAGTC
ACGATTACCGCGGACGAATCCACGAGCACAGCCTACATGGAGCTGAGCAGCCTGAGATCTGAGGACACGGCCGTG
T
ATTACTGTGCGAGAGGAACTGGGGACCACACTACGGTGGTAACTCCTTTCGACTACTGGGGCCAGGGAACCCTGG
T
C
>38++/D-/27+|B1-VH5|Wk52
NNNNNNTAGCTGGTGCAGTCTGGAGCA---
GAGGTGAAGAAGCCTGGGTCCTCGGTGAAGGTCTCCTGCAGGGCTT
CTGGAGGCACCTTC------------
AGCAAGTATGCTATCAGCTGGGTGCGACAGGCCCCTGGACAGGGGCTTGA
GTGGATGGGAGGGATCATCCCTATC------TTTGGTACACCCAATTACGCACAGAAGTTCCAG---

GGCAGAGTC
ACGATTACCGCGGACGAATCCACGAGCACAGCCTACATGGAGCTGAGCAGCCTGAGATCTGAGGACACTGCCGTA
T
ATTACTGTGCGAGAGGAACTGGGGACCACACTACGGTGGTAACTCCTTTCGACTACTGGGGCCAGGGAACCCTGG
T
C
>38++/D-/27+|B4-VH5|Wk52
NNNNNNTAGCTGGTGCAGTCTGGAGCA---
GAGGTGAAGAAGCCTGGGTCCTCGGTGAAGGTCTCCTGCAGGGCTT
CTGGAGGCACCTTC------------
AGCAAGTATGCTATCAGCTGGGTGCGACAGGCCCCTGGACAGGGGCTTGA
GTGGATGGGAGGGATCATCCCTATC------TTTGGTACACCCAATTACGCACAGAAGTTCCAG---
GGCAGAGTC
ACAATTACCGCGGACGAATCCACGAGCACAGCCTACATGGAACTGAGCAGCCTGAGATCTGAAGACACTGCCGTA
T
ATTACTGTGCGAGAGGAACTGGGGACCATACTACGGTGGTAACTCCTTTCGACTACTGGGGCCAGGGAACCCTGG
T
C
>38++/D-/27+|C10-VH5|Wk52
NNNNNNTAGCTGGTGCAGTCTGGAGCA---
GAGGTGAAGAAGCCTGGGTCCTCGGTGAAGGTCTCCTGCAGGGCTT
CTGGAGGCACCTTC------------
AGCAAGTATGCTATCAGCTGGGTGCGGCAGGCCCCTGGACAAGGGCTTGA
GTGGATGGGAGGGATCATCCCTATC------TTTGGTACACCCAATTACGCACAGAAGTTCCAG---
GGCAGAGTC
ACGATTACCGCGGACGAATCCACGAGCACAGCCTACATGGAACTGAGCAGCCTGAGATCTGAGGACACTGCCGTG
T
ATTACTGTGCGAGAGGAACTGGGGACCACACTACGGTGGTAACTCCTTTCGACTACTGGGGCCAGGGAACCCTGG
T
C
