# Supplementary material for: Persistence and selection of an expanded B-cell clone in the setting of rituximab therapy for Sjögren’s syndrome
Source: Arthritis Res Ther. 2014 Feb 11;16(1):R51. doi: 10.1186/ar4481 (PMC3978607; doi:10.1186/ar4481)
Supplement: Additional file 4: Figure S2 — Alignment of the most common clone sequence with germline sequences. Shown is an alignment of the most common clone sequence (upper case) to the most closely matching germline sequences in the ImMunoGeneTics (IMGT) database (lower case). Probable regions of junctional diversification are indicated (n for n-addition and p for p-addition), although somatic hypermutations in the CDR3 sequence cannot be ruled out, since the germline version of the CDR3 sequence is not available for comparison. [file ar4481-S4.pdf]

Fig. S2

```
   VH1-69 nn   D7-27            D 4-23       nn pp J4 02
          CT aactgggga   TG actacggtggtaactcc       gactac tggggccagggaaccctggtcaccgtctcctcag  Putative germline
TGTGCGAGA GG AACTGGGGA CCAC ACTACGGTGGTAACTCC TT TC GACTAC TGGGGCCAGGGAACCCTGGTCACCGTCTCCTCAG  Actual sequence
                       nnnn                   nn pp                                            Non germline encoded
```
