# Supplementary material for: Persistence and selection of an expanded B-cell clone in the setting of rituximab therapy for Sjögren’s syndrome
Source: Arthritis Res Ther. 2014 Feb 11;16(1):R51. doi: 10.1186/ar4481 (PMC3978607; doi:10.1186/ar4481)
Supplement: Additional file 5: Table S3 — List of unique mutations in the large expanded clone from Sjögren’s syndrome subject 2 (SjS2). List of unique mutations found in the 55 sequences in Table S2, organized by position. For each mutation, we list the germline codon it mutated from, its position, the mutant codon the mutated nucleotide is part of, whether that change by itself would be an S or an R mutation, and the number of sequences in which it is found. Note that some codons are mutated in more than one position and so will appear more than once. [file ar4481-S5.pdf]

Table S3

Original codon	position (IMGT)	mutated codon	R or S mutation	No. of sequences
GTG	6	GTA	S	8
CAG	7	GAG	R	13
GGG	15	GGA	S	6
GCT	17	GTT	R	1
GCT	18	GCA	S	7
GAG	24	GAA	S	15
GTG	25	CTG	R	15
GTG	25	TTG	R	1
GTG	27	GTA	S	2
AAG	32	AGG	R	1
CCT	36	CCC	S	1
CCT	36	CCA	S	1
GGG	39	GGA	S	1
AAG	50	AGG	R	1
AAG	51	AAA	S	1
GTC	52	ATC	R	4
TCC	57	TCT	S	1
TGC	60	TGT	S	2
AAG	62	AGG	R	51
AAG	62	AGA	R	2
AAG	63	AGA	R	2
GGA	72	GGC	S	2
GGC	75	GGG	S	2
ACC	78	ACG	S	1
ACC	78	ACA	S	2
ACC	78	ACT	S	4
AGC	96	AGT	S	2
AGC	98	AAG	R	44
AGC	98	AAA	R	10
AGC	98	AAC	R	1
AGC	99	AAG	R	44
AGC	99	AAA	R	10
TAT	101	TCT	R	1
TAT	101	TTT	R	1
GCT	103	TTT	R	1
GCT	104	TTT	R	1
GCT	105	GCC	S	2
ATC	106	CTC	R	1
ATC	106	GTC	R	1
ATC	108	ATT	S	2

AGC	109	GGC	R	1
AGC	110	AAC	R	2
AGC	111	AGT	S	6
GTG	115	ATG	R	1
GTG	115	CTG	R	4
CGA	118	AGA	S	2
CGA	120	CGG	S	3
CAG	123	CAA	S	2
GCC	124	AGT	R	2
GCC	125	AGT	R	2
GCC	126	AGT	R	2
CCT	129	CCC	S	1
CAA	135	CAG	S	14
GGG	136	CGG	R	1
GGG	136	AGA	R	1
GGG	138	GGA	S	1
GGG	138	AGA	R	1
GGG	138	GGT	S	1
GGG	138	GGC	S	1
CTT	139	GTT	R	1
CTT	140	CCT	R	1
GAG	144	GAA	S	4
GGA	153	GGG	S	1
GGG	156	GGA	S	2
ATC	159	ATT	S	2
ATC	161	ACC	R	1
ATC	162	ATT	S	2
CCT	165	CCC	S	3
CCT	165	CCA	S	1
ATC	168	ATA	S	1
ATC	168	ATT	S	4
TTT	177	TTC	S	2
GGT	178	CGT	R	1
GGT	180	GGC	S	1
GGT	180	GGA	S	1
GGT	180	GGG	S	1
ACA	181	GCT	R	1
ACA	183	ACG	S	2
ACA	183	GCT	R	1
GCA	184	CCC	R	40
GCA	184	CCA	R	6
GCA	185	GGG	R	1

GCA	186	CCC	R	40
GCA	186	GGG	R	1
AAC	189	AAT	S	44
TAC	191	TTC	R	1
TAC	192	TAT	S	4
CAG	198	CAA	S	13
CAG	198	CAC	R	1
AAG	200	AGG	R	1
AAG	201	AAA	S	2
TTC	202	ATG	R	1
TTC	204	TTT	S	1
TTC	204	ATG	R	1
CAG	207	CAA	S	1
AGA	216	AGG	S	3
GTC	217	CTC	R	1
ACG	221	ATG	R	1
ACG	221	AAC	R	1
ACG	222	ACC	S	16
ACG	222	ACA	S	6
ACG	222	AAC	R	1
ATT	225	ATA	S	1
ACC	227	AGC	R	2
ACC	228	ACT	S	1
GAA	237	GAC	R	2
GAA	237	GAG	S	2
TCC	240	TCT	S	7
ACG	243	ACA	S	3
AGC	245	ACC	R	5
AGC	245	ACT	R	1
AGC	245	AAC	R	7
AGC	245	AAT	R	1
AGC	246	ACT	R	1
AGC	246	AGT	S	2
AGC	246	AAT	R	1
GCC	250	TCC	R	1
GCC	252	GCG	S	1
TAC	253	CAC	R	1
TAC	255	TAT	S	1
ATG	256	TTG	R	6
GAG	261	GAA	S	40
CTG	262	GTG	R	4
CTG	262	ATG	S	1

CTG	262	TTG	R	1
AGC	265	GGC	R	1
AGC	266	AAC	R	1
AGC	267	AGT	S	4
AGC	268	GGC	R	2
AGC	270	AGT	S	2
CTG	273	CTC	R	21
CTG	273	CTA	R	2
AGA	275	ACA	R	8
AGA	275	AAA	R	1
GAG	282	GAA	S	26
GAG	282	GAT	R	1
GAC	285	GAT	S	1
ACG	288	ACT	S	37
ACG	288	ACA	S	3
GTG	292	ATA	R	8
GTG	292	TTG	R	1
GTG	294	GTC	S	3
GTG	294	GTA	S	22
GTG	294	ATA	R	8
GTG	294	GTT	S	9
TAT	297	TAC	S	5
TAC	299	TTC	R	1
TAC	300	TAT	S	5
TGT	303	TGC	S	1
AGA	307	CGA	S	2
ACT	314	AGT	R	1
ACT	315	ACG	S	1
ACT	315	ACA	S	1
CAC	324	CAT	S	7
ACT	327	ACC	S	3
ACT	327	ACA	S	4
ACG	330	ACA	S	1
GTG	333	GTA	S	2
GTA	334	CTA	R	1
GTA	336	GTG	S	4
GTA	336	GTC	S	1
GTA	336	GTT	S	1
ACT	338	ATT	R	1
ACT	339	ACC	S	1
CCT	342	CCC	S	1
GAC	348	GAT	S	1

TAC	350	TTC	R	1
TAC	351	TAT	S	2
CAG	359	CCG	R	1
CAG	360	CAA	S	5
GGA	363	GGC	S	7
CTG	369	CTA	R	1
