# Supplementary material for: Persistence and selection of an expanded B-cell clone in the setting of rituximab therapy for Sjögren’s syndrome
Source: Arthritis Res Ther. 2014 Feb 11;16(1):R51. doi: 10.1186/ar4481 (PMC3978607; doi:10.1186/ar4481)
Supplement: Additional file 6: Table S4 — Analysis of somatic hypermutation selection in other expanded clones. Following the same format as Table 2, this table summarizes the selection in all of the clones identified in our experiments. Overall they exhibit negative selection, much like the large VH1-69 clone in Sjögren’s syndrome subject 2 (SjS2). However, their small numbers weaken the strength of detection. [file ar4481-S6.pdf]

Table S4

clones with one sequence

Type	ID	Observed Mutations				Expected				Focused Test	
		CDR		FRW		CDR		FRW		P-value	
		R	S	R	S	R	S	R	S	CDR	FRW
Germline|HHIGHV3-30_18|HHIGHJ5_01	38++/IGD-/27+|G2-VH3	7	0	11	4	0.176	0.041	0.563	0.22	0.0598	0.385
	38++/IGD-/27+|G5-VH3	7	2	11	4	0.176	0.041	0.563	0.22	0.157	-0.338
Germline|HHIGHV1-2_02|HHIGHJ4_02	38++/D-/27+|E4-VH1/5	1	1	4	5	0.143	0.047	0.595	0.215	-0.144	-0.0245
	38++/D-/27+|H11-VH1/3/5	2	1	5	4	0.174	0.057	0.572	0.197	-0.288	-0.0902
Germline|HHIGHV3-30_02|HHIGHJ6_03	38-/D-/27+|F2-VH1/3	2	0	3	2	0.178	0.045	0.555	0.222	0.328	-0.322
	38-/D-/27+|G7-VH1/3	2	0	3	1	0.178	0.045	0.555	0.222	0.176	0.435
Germline|HHIGHV1-2_04|HHIGHJ3_02	38+/IGD+/27+|A11-VH1	4	1	8	7	0.144	0.047	0.595	0.215	-0.479	-0.046
	38+/IGD+/27+|A11-VH5	4	1	8	7	0.144	0.047	0.595	0.215	-0.479	-0.046
Germline|HHIGHV4-59_01|HHIGHJ6_02	38+/IGD+/27+|A9-VH4	4	3	11	1	0.165	0.041	0.556	0.238	0.216	0.332
	38+/IGD+/27+|A9-VH6	4	3	11	4	0.166	0.041	0.555	0.237	0.495	-0.284
Germline|HHIGHV3-23_04|HHIGHJ3_01	38++/D-/27+|B10-VH3	8	2	4	3	0.171	0.047	0.556	0.226	0.0458	-0.0745
	38++/D-/27+|D2-VH3	8	2	4	2	0.171	0.047	0.556	0.226	0.0246	-0.144

clones with more than one sequence

	Observed Mutations				Expected				Focused Test	
	CDR		FRW		CDR		FRW		P-value	
	R	S	R	S	R	S	R	S	CDR	FRW
SjS1_Wk 36_HHIGHV3-30_18_HHIGHJ5_01_367	10	2	13	6	0.245	0.063	0.498	0.194	0.283	-0.323
SjS4_Wk 52_HHIGHV1-2_02_HHIGHJ4_02_388	6	1	5	6	0.261	0.085	0.481	0.173	-0.385	-0.0459
SjS4_Wk 52_HHIGHV3-30_02_HHIGHJ6_03_379	4	1	3	2	0.279	0.077	0.461	0.184	0.397	-0.225
SjS2_Wk 0_HHIGHV1-2_04_HHIGHJ3_02_364	4	1	8	7	0.207	0.064	0.536	0.193	-0.232	-0.0651
SjS2_Wk 0_HHIGHV4-59_01_HHIGHJ6_02_370	4	3	13	4	0.268	0.073	0.462	0.197	-0.193	0.462
